# Chemotherapy and tumor microenvironment of pancreatic cancer

**DOI:** 10.1186/s12935-017-0437-3

**Published:** 2017-07-05

**Authors:** Qiaofei Liu, Quan Liao, Yupei Zhao

**Affiliations:** Department of General Surgery, Peking Union Medical College Hospital, Peking Union Medical College & Chinese Academy of Medical Sciences, 1# Shuai Fu Yuan, Dong Dan District, Beijing, 100730 China

**Keywords:** Pancreatic cancer, Tumor microenvironment, Chemotherapy, Myeloid derived suppressor cells, Tumor associated macrophages, Pancreatic stellate cells, Cancer associated fibroblasts

## Abstract

Pancreatic cancer is an extremely dismal malignance. Chemotherapy has been widely applied to treat this intractable tumor. It has exclusive tumor microenvironment (TME), characterized by dense desmoplasia and profound infiltrations of immunosuppressive cells. Interactions between stromal cells and cancer cells play vital roles to affect the biological behaviors of pancreatic cancer. Targeting the stromal components of pancreatic cancer has shown promising results. In addition to the direct toxic effects of chemotherapeutic drugs on cancer cells, they can also remodel the TME, eventually affecting their efficacy. Herein, we reviewed the following four aspects; (1) clinical landmark advances of chemotherapy in pancreatic cancer, since 2000; (2) interactions and mechanisms between stromal cells and pancreatic cancer cells; (3) remodeling effects and mechanisms of chemotherapy on TME; (4) targeting stromal components in pancreatic cancer.

**Electronic supplementary material:**

The online version of this article (doi:10.1186/s12935-017-0437-3) contains supplementary material, which is available to authorized users.

## Background

Pancreatic cancer is always referred to pancreatic ductal adenocarcinoma (PDAC) which is the fourth leading cancer death in USA. Its recent 5-year overall survival of pancreatic cancer is only 7.7% and its median survival time is about 6 months [1]. Chemotherapy is one of the most important treatments for patients with advanced pancreatic cancer. Several clinical advances of chemotherapy have been achieved by high quality, large scale, prospective and randomized clinical trials. Adjuvant chemotherapy based on gemcitabine or fluorouracil have shown promising effects to improve the overall survival [2, 3]; oral fluorouracil, S-1, has been reported to show better results than gemcitabine [4]; palliative FOLFIRINOX (oxaliplatin, irinotecan, fluorouracil, and leucovorin) regimen was reported to be the best choice for patients with metastatic pancreatic cancer [5]. For some selected borderline or local unresectable pancreatic cancer, neoadjuvant chemotherapy have also been initially adopted, with the hope to lower down the tumor and regain the radical resection opportunities [6, 7].

Increasing interests have been put into approaches targeting the tumor stroma of pancreatic cancer. The TME of pancreatic cancer is characterized by dense desmoplasia and extensive immunosuppression [8]. Pancreatic stellate cells (PSCs) and cancer associated fibroblasts (CAFs) are the main matrix-producing cells in TME of pancreatic cancer [9]. Tumor associated macrophages (TAMs) and myeloid derived suppressor cells (MDSCs) are the most infiltration populations of immunosuppressive cells in the TME [10]. The network consisting of stromal cells and cancer cells has become to be the most shining star in the research field of pancreatic cancer. Targeting the stromal components has also shown primary positive results in pancreatic cancer [11–14].

Interactions between chemotherapy and TME have also been paid more and more attentions. On one hand, chemotherapy can induce immunogenic cell death (ICD) in certain tumors, which could potentially activate immune system. On the other hand, these chemotherapeutic drugs can also remodel the TME. Gemcitabine was reported to inhibit the expansion of MDSCs [15], however, it was also reported to induce T helper 2 (Th2) cytokine environment in TME which induce the polarization of M2 polarized TAMs [16]. After gemcitabine treatment, pancreatic cancer secreted more GM-CSF, recruiting MDSCs to diminish the efficacy [17]. Cisplatin or carboplatin increased the potency of tumor cell lines to secrete interleukin (IL)-6 and prostaglandin E2 (PGE2) to induce IL-10-producing M2 polarized TAMs [18].

Four aspects focusing on the chemotherapy and TME of pancreatic cancer were reviewed in this paper, including: clinical landmark advances of chemotherapy in pancreatic cancer, since 2000; interactions and mechanisms between the stromal cells and pancreatic cancer cells; remodeling effects and mechanisms of chemotherapy on TME; targeting of the stromal components in pancreatic cancer.

## The advances of chemotherapy in pancreatic cancer, since 2000

In respect of adjuvant chemotherapy, in 2001 and 2004, two papers substantially demonstrated that fluorouracil based adjuvant treatment improved overall survival, however chemoradiotherapy showed no survival benefits [2, 19]. In 2007, Oettle et al. [20] reported postoperative gemcitabine improved the estimated disease free survival at 3 and 5 years. In 2010, Neoptolemos et al. reported adjuvant use of fluorouracil plus folinic acid had comparable results with gemcitabine [3]. In 2013, adjuvant use of gemcitabine was reported to improve the 5-year overall survival and 10-year overall survival [21]. In 2016, Uesaka et al. revealed that adjuvant use of oral fluorouracil (S-1) achieved 44.1% of 5-year overall survival. Recently, Neoptolemos et al. [22] reported that the combinational use of gemcitabine with capecitabine prolonged the median survival of patients with resected pancreatic cancer.

In 2011, Conroy et al. [5] reported that for the patients with metastatic pancreatic cancer, FOLFIRINOX regimen significantly improved the results compared with gemcitabine alone. FOLFIRINOX improved median progression-free survival (PFS) and overall survival compared with gemcitabine alone. In 2013, the combination of nab-paclitaxel with gemcitabine was reported to significantly increase the response rate, improved PFS and overall survival among patients with metastatic pancreatic cancer, compared to gemcitabine alone [23]. In 2014, OFF (oxaliplatin, folinic acid and fluorouracil) was demonstrated to have better results than FF (folinic acid and fluorouracil) alone in patients with advanced gemcitabine refractory pancreatic cancer [24]. In 2016, Wang-Gillam et al. [25] reported nanoliposomal irinotecan in combination with fluorouracil and folinic acid significantly extended survival in patients with metastatic pancreatic cancer who previously received gemcitabine based therapy.

Theoretically, neoadjuvant therapy has several potential advantages over adjuvant therapy including better drug absorption, assessment of response, improved resectability rate and increased margin-negative resection rate [26]. However, the effects of neoadjuvant therapy in pancreatic cancer have not been confirmed. In 2010, a meta-analysis, mainly based on retrospective data, reported that approximately 30% of initially non-resectable tumor patients would be expected to have resectable tumors after neoadjuvant therapy, with comparable survival as initially resectable tumor patients [26]. In 2015, Ferrone et al. [6] reported neoadjuvant FOLFIRINOX for the patients with borderline resectable pancreatic cancer, resulted in a significant decrease in tumor size, lower morbidity, lymph node positivity, perineural invasion and overall survival. For the patients with resectable pancreatic cancer, due to the consideration of the risk of disease progression after neoadjuvant treatment, the clinical trials of neoadjuvant treatment is considered to be difficult and some perspective clinical trials were terminated early due to slow recruiting [7]. (The chronological list of clinical landmark events of chemotherapy in pancreatic cancer from 2000 is shown in Additional file 1: Table S1).

## Interactions and mechanisms between stromal cells and pancreatic cancer cells in TME

Pancreatic cancer is a well-known inflammatory malignance. It has exclusive pathological characteristics, with an extensive desmoplastic stroma and immunosuppressive environment, comprised of abundant cellular components, mainly including PSCs, CAFs, TAMs and MDSCs. The cancer cells only consist of approximately 10–30% of the cellular components. Interactions between the cancer cells and the TME components facilitate tumor initiation, progression, metastasis and resistance to chemotherapy by varieties of mechanisms. Herein, we summarized eight potential tumor-supporting mechanisms contributing to the malignant behaviors of pancreatic cancer, through the interactions between cancer cells and the stromal cells in TME, including: (1) maintenance of pancreatic cancer stem cells (PCSCs); (2) modeling of the extracellular matrix (ECM); (3) promotion of the proliferation and survival of cancer cells; (4) promotion of the migration of cancer cells; (5) promotion of epithelial–mesenchymal transition (EMT); (6) promotion of the angiogenesis; (7) promotion of lymphangiogenesis; (8) induction of immunosuppressive reactions.

### PSCs

More than 80% of the human pancreatic cancer tissue is the highly desmoplastic stroma. The principal cells responsible for the production of this stroma are PSCs in pancreatic cancer. In the healthy pancreas, the PSCs are always in quiescent status. These qPCSs have stellate shape and express desmin, nestin, vimentin, and glial fibrillary acid protein (GFAP) and exhibit abundant vitamin A containing lipid droplets in their cytoplasm. When activated, they will lose lipid droplets and develop a spindle-shaped morphology, express α-smooth muscle actin (α-SMA), proliferate, migrate, and secrete excessive amount of ECM proteins, leading to the imbalance between ECM production an degradation and eventually extensive desmoplasia.

A large number of interleukins (IL-1, IL6 and IL10), chemokines (C-X3-C motif chemokine ligand 1, CX3CL1) growth factors (e.g., vascular endothelial growth factor, VEGF; platelet-derived growth factor, PDGF; transforming growth factor beta, TGFβ) and tumor necrosis factor alpha (TNFα), have been identified to activate qPSCs [27]. Recently, Bhatia et al. [28] reported that pancreatic parathyroid hormone related protein (PTHrP) secreted by islet cells can also activate qPSCs. The aPSCs can proliferate, migrate to the injured location, with expression of α-SMA, changes of morphology and secretion of ECM proteins. These different processes are controlled and regulated by varieties of signal pathways (Table 1) [9]. The PSCs may be even activated at the pretumoral lesions and reciprocally promote cancerogenesis. Pando et al. [29] reported that a distinct stromal reaction and aPSCs around pancreatic intraductal neoplasia (PanIN) lesions which led to pancreatic cancer in a pancreatic cancer murine model overexpression KrasG12D. Cocultured with PanIN cells isolated from KrasG12D mice significantly increased proliferation, activation and ECM production of PSCs [30].

**Table 1:** The signal pathways to regulate the biological behaviors of PSCs

	Proliferation	Migration	ECM production
Hedgehog		+	
JAK-STAT	+		
MAPK	+	+	+
PI3K	+	+	+
PKC			+
Rho kinase			+
Smads			+
Wnt/β-catenin	+		+
PPAPγ	+		
TF(AP-1, NK-κB, Gli-1)	+	+	+

Some subpopulations of PSCs have been reported to have different roles in pancreatic cancer. Ikenaga et al. reported that the frequency of CD10 expression by PSCs was markedly higher in tumor tissue than in normal tissue (33.7% versus 0%). CD10(+) PSCs was associated with positive nodal metastases and a shorter survival time. These CD10(+) PSCs secreted more MMP3 and increased the invasion and growth of pancreatic cancer cells [31]. Fujiwara et al. [32] reported that CD271(+) PSCs seemed to appear at the early stage of pancreatic carcinogenesis and that CD271 expression was significantly correlated with a better prognosis in patients with pancreatic cancer.

### CAFs

CAFs are also the main source of the collagen-producing cells in varieties of cancers. Unlike the stellate cells, which are exclusively located in liver and pancreas, CAFs are widely located in many normal tissue and tumor tissues. Many markers have been proposed to detect CAFs in different tissues, including α-SMA, tenascin-C, fibroblast activation protein (FAP), thy-1 (CD90), podoplanin, vimentin, fibronectin, type I collagen, prolyl4-hydroxylase, and fibroblast specific protein-1 (FSP-1)/S100A4 [33–35]. However, none of these markers are exclusively expressed on CAFs. A combination of morphological appearance and a marker definition are the most reliable methods to detect CAFs. CAFs in pancreatic cancer are another main effector cell population contributing to the desmoplasia. The originations of CAFs in pancreatic cancer include resident fibroblast, bone marrow derived cells, and PSCs [36]. Resident fibroblasts express α-SMA but do not express neural markers, such as nestin and GFAP, which is different from PSCs. After injuries of pancreas, inflammatory cytokines and chemokines from inflammatory cells, endothelial cells, or cancer cells activate resident fibroblasts and they proliferate and differentiate into CAFs [37, 38].

High intratumoral infiltration of several subtypes of CAFs, such as podoplanin, FAP or CD90 positive CAFs, predicted poorer prognosis of colon cancer, breast cancer, lung cancer and prostate cancer [35, 39–41]. Although the tumor-promoting roles of CAFs have been widely recognized, some studies also reported the tumor suppression by CAFs. In early stage of colon cancer, secretion of TGF-β by CAFs suppressed tumor initiation, however, TGF-β promoted cancer development in the advanced stage [42]. Flaberg et al. [43] reported that CAFs inhibited proliferation of cancer cell lines in vitro. Podoplanin-expressing CAFs inhibit growth of small cell lung cancer cells possibly under direct contact [44]. The dual roles of CAFs may be cancer cell type-dependent and may be changeable during the different stages of cancer.

### TAMs

Inflammation is now a well-recognized hallmark of varieties of malignancies and pancreatic cancer is one of the most well-known inflammatory cancers. In a cancerogen, DMBA (dimethylbenzanthracene)-induced murine pancreatic cancerogenesis model, with the progression of tumor initiation, the proportion of CD45 positive inflammatory cells rising from 15.5% in normal pancreatic tissue, to more than 50% in tumor tissue. The percentages of MDSCs, TAMs and the ratio of M2/M1 were significantly elevated with the progression of pancreatic cancerogenesis, in contrast, the percentages of helper T cell and cytotoxic T cell were significantly decreased. TAMs were one of the prevalent inflammatory cells in the TME [10, 45].

It is generally believed that tissue macrophages originate from circulating monocytes which extravasate into the tissues and then differentiate into mature macrophages, under the inductions of the tissue signals. However, recent studies showed that besides of the circulating monocytes, tissue resident macrophages can originate from yolk sac and fetal liver [46]. Most of the resident macrophages in skin, spleen, pancreas, liver and peritoneum cavity originate from yolk sac progenitors or fetal liver and are maintained independent from circulating monocytes. However, resident macrophages in gut only originate from circulating monocytes, and the resident macrophages in lung and kidney have dual origins [47, 48]. In contrast, macrophages involved in pathological responses appear to mainly come from circulating bone marrow derived monocytes [49]. Variable soluble factors have been reported to recruit the monocytes rom peripheral blood. Colony stimulating factor-1 (CSF-1) is abundantly expressed by many tumor cells and their stromal cells in TME. Tumor microenvironment-derived CSF-1 is the mast regulator of recruitment and differentiation of circulating monocytes, and knockout of CSF-1R showed depletion of TAMs [50, 51]. Another CSF-1R, IL-34 also showed to recruit TAMs [52]. In a xenograft model of skin cancer, VEGFA recruited monocytes to differentiate into TAMs [53]. Some chemokines, including CCL2, CCL18, CCL9, were reported to recruit the ly6c(+) monocyte into the tumor microenvironment in murine breast cancer and colon cancers [54–56]. Angiotensin-II was found to be responsible for the amplification of the self-renewing progenitor cells and hence the production of TAMs [57].

Plasticity and diversity are hallmarks of TAMs. Once the circulating monocytes are recruited into the tumor microenvironment, they will be induced to diverse phenotypes by various signals, including hypoxia, metabolic products, tissue damage, growth factors, cytokines, and chemokines. TAMs secrete varieties of cytokines, chemokines, poly-peptide growth factors, hormones, MMPs and metabolites, most of which possess tumor-promoting activities [58–60]. Description of macrophage activation has been currently contentious and confusing. In 1990s, differential effects of IL-4 or IL-13 compared to IFN-γ and/or lipopolysaccharide (LPS) on macrophage gene expression were described. The macrophages activated by IL-4 or IL-13 were termed to be “alternative activation,” and the ones activated by IFN-γ and/or LPS were termed to be “classical activation” [61]. Mill et al. proposed the terminology M1 for classical activated macrophages, and M2 for alternative activated macrophages in 2000 [62]. M2 was further defined into M2a, M2b, M2c for different activation scenarios [63]. Diversity of terminology of macrophages activated by different signals have impeded researches significantly. To tackle this issue, an international consensus nomenclature system was proposed in 2014 [64]. M1- and M2-polarized TAMs are only extremes of a continuum in a universe of functional states and most of the TAMs are in the continuum changeable status between M1 and M2 [65]. Activation of TAMs from tumor microenvironment of various tumors include hypoxia [66, 67], metabolic products of cancer cells (e.g., lactic acid) [59, 68], COX-2 [69], cytokines (e.g., TGF-β, CSF-1, GM-CSF), interleukins (IL-4, IL-10, IL-13) and plasma cells and immune complexes, damage associated molecular patterns (DAMPs), such as high-mobility group box1 protein (HMGB1), extracellular ATP, and degraded extracellular matrix components produced by cancer cells or stromal cells [70]. Signal pathways involved in M1 polarization include NF-Kappa B, STAT1, and IRF5, whereas IRF4, STAT6, MYC, PPARγ and KLF4 have been reported to promote M2 polarization [46, 65, 71]. Once activated, TAMs exert different functions to affect the malignant behaviors of cancers, which predominantly promote the invasiveness of cancer cells. The high infiltrations of TAMs, especially the M2 polarization TAMs, in tumor tissue predicted poor prognosis of many cancers, including pancreatic cancer.

### MDSCs

The phenomenon that tumor can induce myelopoiesis has been observed for more than 100 years [72]. During myelopoiesis, various immature myeloid cells were generated, which lack of expressions of specific terminated markers for T cells, B cells, dendritic cells, NK cells and macrophages, and for a long time, these cells were called null cells. In 1960s, these cells were reported to induce a leukaemoid reaction which promoted tumor growth [73]. For a long time, owing to the phenotypic heterogeneity without a consensus regarding the cellular phenotype of these cells, diverse nomenclature, including immature myeloid cells (iMCs), myeloid suppressor cells (MSCs) and GR1(+) myeloid cells were recommended. Until 2007, a consensus reached to nominate MDSCs as the term for these cells [74].

In mice, MDSCs are generally defined as GR-1(+)CD11b(+) cells. And further, the murine MDSCs consist of two subgroups with different mononuclear and polymorphonuclear morphology and surface markers. Polymorphonuclear MDSCs (PMN-MDSCs) are referred to CD11b(+)Ly6C(low)Ly6G(+) cells and mononuclear MDSCs (M-MDSCs) were referred to CD11b(+)Ly6C(high)Ly6G (−) cells and M-MDSCs have potential to differentiate into terminated macrophages and dendritic cells. More than 80% of MDSCs are PMN-MDSCs [75, 76]. In humans, the definitions of human MDSCs are more complicated. Historically, human MDSCs were defined as lineage markers and HLA-DR(−), and CD33(+) cells purified with mononuclear cells on ficoll gradient. PMN-MDSCs are characterized as CD11b(+)CD14(−)CD15(+) or CD66b(+). M-MDSCs are defined by CD14(+)HLA-DR(low). As well, PMN-MDSCs represent majority of the MDSCs in human cancer patients [77, 78].

It should be noted that in the bone marrow of normal mouse, there are also some cells with identical phenotype of MDSCs, however these cells do not have immunosuppressive capacities. So, MDSCs should also be activated to exert functions. Theory of “two sets of signals” has been proposed for the expansion and activation of MDSCs. The first set of signals promotes the expansion of MDSCs from bone marrow, and the second set of signals activates MDSCs [79]. The first set of these signals are regulated largely by GM-CSF, M-CSF, G-CSF, and other growth factors produced by tumor cells and tumor stromal cells [80, 81]. Then the second set of signals activate MDSCs, mainly by prostaglandin E2(PGE2), IL-1β, IL-4, IL-6, IL-10, IL-13, VEGF and TGF-β [82–85]. Recent studies reported that HMGB1 and PPARγ can also activate MDSCs by activation of STAT3, NF-κB, Erk1/2, and p38 signal pathways. Members of the STATS (STAT3, STAT5, and STAT6) have been considered to be critical factors in the regulation of MDSCs expansion and activities [86–88]. The downstream targets included S100A8, S100A9 and C/EBPβ [89]. Some chemokines are involved in the recruitment of MDSCs into tumor tissue. CXCL1, CXCL2, and CXCL5 have been shown to recruit MDSCs by binding to CXCR2. CXCL12 can also recruit MDSCs, by binding to CXCR4 [90].

After the process of expansion, recruitment and activation, in addition to immunosuppression, MDSCs exert various functions to promote the initiation, progression, and metastasis of cancers. Many mechanisms of immune suppression induced by MDSCs have been proposed, including production of ARG1, iNOS, IL-10, TGF-β, COX2 and induction of Tregs [90]. M-MDSCs and PMN-MDSCs were reported to exert different mechanisms of immune suppressions. M-MDSCs can suppress both antigen-specific and nonspecific T cell responses and show stronger suppressive activities than PMN-MDSCs. M-MDSCs exert immunosuppression through production of NO, however, PMN-MDSCs mainly depended on ROS. Both of them use ARG-1 for their suppressive activities [76, 91]. Peroxynitrite (PNT), the production of NO and superoxide, can inhibits T cells by nitrating T cell receptors (TCRs) which reduces their binding to cognate antigen-MHC complexes [92]. Depletion of l-arginine and cysteine by ARG-1 caused by MDSCs resulted in decreased CD3ζ chain expression, leading to reduction of IL-2 and IFN-γ to inhibit T lymphocyte proliferation. Several studies also showed that M-MDSCs could induce or recruit FOXP3+ Treg cells by different mechanisms, including production of TGF-β, CCR5 and ARG-1 [93, 94]. MDSCs have also been suggested to have a role in tumor angiogenesis in some tumors [95, 96]. Hypoxia can promote MDSC migration into tumor site via HIF-1α-induced production of chemokines and the recruited MDSCs will secret VEGF, basic fibroblast growth factor (bFGF) through activation of STAT3 to promote angiogenesis [97]. Bombina variegata peptide8 (Bv8) can also be induced by STAT3 to promote angiogenesis then enhance lung metastasis [98]. MDSCs were also reported to secret MMP-9 to promote tumor angiogenesis [90]. PMN-MDSCs produced HGF and TGF-β to induce EMT of primary melanoma cells. MDSCs can induce cancer stem cells of ovarian cancer by upregulation of microRNA-101 to target CtBP2 [99]. Circulating tumor cells (CTC) derived from the primary cancer initiate distant metastasis by entering and traversing the bloodstream. MDSCs have potential to direct interact with CTCs to form cell-cluster to promote metastasis [100]. MDSCs accumulated in the PanIN lesions in the DMBA-induced and genetically defined pancreatic cancerogenesis murine model [45]. With progression of pancreatic cancerogenesis, the proportions of MDSCs in total inflammatory cells in pancreatic lesions increased from 5.24% in normal pancreatic tissue, 9.25% in low grade PanIN, 15.25% in high grade PanIN to 22.34% in invasive pancreatic cancer [10]. In addition, increasing MDSCs in peripheral blood of pancreatic cancer patient was associated with increased risk of death, and it was an independent prognostic factor for survival [101].

In Table 2, we systematically presented the advances of the roles and mechanism of these stromal cells to regulate the malignant behaviors of pancreatic cancer in eight tumor-supporting aspects in detail during the last several years.

**Table 2:** Roles and mechanisms of the stromal cells in TME of pancreatic cancer

	Maintenance of PCSCs	Modeling of ECM	Proliferation and survival	Migration
PSCs	1. PSCs secreted-IL-6 stimulates STAT3 to enhance colony formation and progression of PanIN [102] 2. PSCs enhance the CSCs phenotype of cancer cells by TGF-β [103] 3. PSCs promote sphere formation by paracrine Nodal/Activin signaling [104] 4. PSCs enhance the spheroid-forming of cancer cells and induces the expression of CSC related genes ABCG2, Nestin and LIN28 [105]	1. Hypoxic PSCs exhibit highly organized parallel patterned matrix fibers to promote cancer cell motility by inducing directional migration via PLOD2 [106] 2. PSC-derived collagen I induces haptokinesis and haptotaxis of cancer cells by activating FAK signaling via binding to integrin α2β1 [107] 3. PSCs promote invasion of cancer cells by secretion of MMP3 [108] 4. TGF-β inhibits the secretion of lumican in PSCs, which could enhance PSCs adhesion and mobility [109] 5. PSCs modulate 3D collagen alignment to promote the migration of cancer cells [110]	1. PSCs induce cancer cell proliferation via galectin-1 [111] 2. PSCs improve the survival of cancer cell by supporting the metabolism through autophagic alanine secretion [112] 3. PSCs promote the proliferation of cancer cells via β1-integrin [113] 4. PSCs promote the proliferation of cancer cells by secreting kindlin-2 [114] 5. Autophagic PSCs produce ECM molecules and IL6 to promote the proliferation and invasion of cancer cells [115]	1. PSCs promote the migration of cancer cells via EMT process [116] 2. PSCs promote the migration and invasion of cancer cells via Stromal Cell-Derived Factor-1/CXCR4 Axis [117] 3. PSCs can stimulate the proliferation, migration and chemokine (C-X-C motif) ligands 1 and 2 in pancreatic cancer cells by secreting exosome [118]
CAFs	1. Pancreatic cancer cells-induced expression of miR-21 in CAF promotes the clonogenicity and pancreatoshpere formation [119, 120]	1. CAFs can secrete components of the ECM and change the structure of the ECM via MMPs and β1-integrin [121] 2. FAP expressing fibroblasts remodel the ECM to enhance directionality and velocity of pancreatic cancer cells by beta1-integrin/FAK signal pathway [122] 3. CAF-secreted SPARC maintain the vascular basement membrane to inhibit the metastasis of pancreatic cancer cells [123]	1. FAP expressing fibroblasts inactivate retinoblastoma (Rb) protein in pancreatic cancer cells to promote the proliferation [124] 2. Pancreatic cancer cell induced-SOCS1 gene methylation in CAF activates STAT3 and IGF-1 expression to support growth of pancreatic cancer [125] 3. CAF-drived CXCL12 promotes proliferation of cancer cells by binding CXCR4 [126] 4. Gemcitabine treatment can increase release the exosome of CAF to promote proliferations of cancer cells through Snail [127]	1. CAFs stimulate the migration of PDAC cells through paracrine IGF1/IGF1R signaling [128] 2. CAFs promote migration of pancreatic cancer cells by secreting extracellular vesicles, ANXA6/LRP1/TSP1 [129] 3. CAFs promote the migration and EMT of pancreatic cancer cells via IL-6 [130] 4. Pancreactic cancer cell-induced low expression of CD146 in CAF promoted migration and invasion of cancer cells [131]
TAMs	1. Pancreatic cancer potentially recruits protumoral TAMs by GM-CSF and then TAMs maintain the PCSCs by IL-6/STAT3 signaling pathway [132, 133]	1. Cancer cell derived-CCL2 induced by HIF-1 recruits TAMs to activate PSC to remodel the ECM [134] 2. TAMs secrete granulin to activate hepatic stellate cells, resulting in a fibrotic environment to promote liver metastasis of pancreatic cancer [135] 3. The interactions of TAMs and PSCs contribute the fibrogenesis during pancreatic cancerogenesis [136]	1. TAMs induced-upregulation of CDA improves the survival of cancer cells when treated by gemcitabine [137] 2. Pancreatic cancer cells can secret lectin Reg3 beta to promote M2 through STAT3 singnal pathway and then M2 can inhibit apoptosis and prolong the viability of cancer cells [138]	1. TAMs secrete glial-derived neurotrophic factor, inducing phosphorylation of RET and downstream activation of extracellular signal-regulated kinases (ERK) to promote migration of cancr cells [139] 2. Soluble factors from cancer cells trigger scavenger receptor A on TAMs to promote migration of cancer cells [140] 3. Cancer cell over expressed heparanase induce procancerous phenotype of macrophage to promote migration of cancer cells via IL6/STAT3 signal pathway [141]
MDSCs	1. Pancreatic cancer can induce MDSCs by STAT3 signal pathway and MDSCs increase the ALDH(+)PCSCs [142]	–	1. Pancreatic cancer cells can induce MDSCs that promote tumor cell survival and accumulation [143]	–

	EMT	Angiogenesis	Immunosuppression
PSCs	1. PSCs decrease the expression of E-carderin and ZO-1, increase the expression of β-catenin and vimentin in pancreatic cancer cells [116, 144] 2. IL-6 from PSCs promote EMT in PDAC cells via Stat3/Nrf2 pathway [145]	1. PSCs accompany cancer cells to metastatic sites, stimulate angiogenesis, and are able to intravasate/extravasate to and from blood vessels [146] 2. Heptocyte growth factor (HGF)/c-Met pathway plays a role in PSC-induced tube formation of endothelial cells formation of human microvascular endothelial cells [147] 3. PSCs express both pro- and anti-angiogenic factors to maintain the balance of angiogenesis [148]	1. PSCs induce apoptosis and anergy of T cells via galectin-1 [149, 150] 2. PSCs induce MDSCs via IL-6/JAK/STAT3 signaling axis [151, 152] 3. PSCs can sequester CD8+ T cells by interaction between CXCL12 and CXCR4 [153] 4. PSCs activate mast cells to produce IL13 and tryptase, stimulating proliferation of both cancer cells and PSCs [154]
CAFs	1. CAF-drived CXCL12-CXCR4 signal promotes pancreatic cancer cell EMT and invasion by activating the P38 pathway [155] 2. CAFs promote EMT of pancreatic cancer cells via IL-6/PI-3 signal pathway [156]	1. CAFs potentially induce angiogenesis by CXCL12/CXCR4 axis and SPARC [157]	1. CAFs induce immunosuppressive environment to dampen the effects of antibodies against CTLA-4 and PD-L1 by CXCL12 [12] 2. CAFs weaken the function and survival of T cells by arginase II [158] 3. CAFs induce apoptosis of T cells by galectin-1 [159] 4. CAFs can induce M2 by secreting M-CSF to promote the pancreatic tumor cell growth, migration, and invasion [160]
TAMs	1. M2-polarized TAMs promote EMT in pancreatic cancer cells, partially via TLR4/IL-10 signaling pathway [161] 2. Both M1 and M2-polarized TAM decrease expression of E-cadherin and increase expression of vimentin [162]	1. TAMs potentially induce angiogenesis by secreting VEGF to promote metastasis of pancreatic cancer [163]	1. Blockage of CSFR reprograms TAM s to an antigen-presenting phenotype and improves antitumor T cell responses [164] 2. TAMs potentially induce Treg to promote metastasis of pancreatic cancer [163] 3. Radiation induced M-CSF in cancer cells recruits TAMs to construct a immunosuppressive environment to hamper antitumor response [165] 4. Ly6C(low)F4/80(+) macrophages outside of the tumor microenvironment regulate infiltration of T cells into tumor tissue and establish a site of immune privilege [166]
MDSCs	–	–	1. The MDSCs in pretumroal and pancreatic cancer tissue have high arginase activity and suppress T-cell responses [167] 2. Pancreatic cancer induces bone marrow mobilization of MDSCs to promote tumor growth by suppressing CD8(+) T cells [168] 3. PAUF enhance the immunosuppressive function of MDSCs via the TLR4-mediated signaling pathway [169] 4. Pancreatic cancer dampens SHIP-1 to expand MDSCs and enhance the immunosuppressive functions [170] 5. Depletion of Gr-MDSC, can unmask an endogenous T cell response, disclosing an unexpected latent immunity against pancreatic cancer [143]

## Chemotherapy and tumor microenvironment

Chemotherapy is one of the main modalities for many advanced solid malignancies. However, most of the malignancies showed resistance to chemotherapy. The mechanisms of the resistance largely remain unknown. During the last several decades, the overwhelming attentions have been focused on cancer cells. However, the possible roles of tumor microenvironment in regulation the efficacy of chemotherapy have been largely neglected. On one hand, chemotherapy can direct kill or damage cancer cells, on the other hand, the chemotherapeutic drugs can also remodel the TME. For some tumors, chemotherapy could lead to immunogenic death of cancer cells and then triggered the anti-tumor immunities by activation of T cells, NK cells or macrophages. However, in contrast, chemotherapy has been also reported to remodel tumor microenvironment which promotes tumor regrowth and drug resistance. Herein, we summarized the pro-tumoral effects and anti-tumoral remodeling effects of different chemotherapeutic drugs on TME (Additional file 1: Table S2).

### Conventional cytotoxic chemotherapy

After treatment with these cytotoxic drugs, the damage of the tumor tissue could be repaired to a tumor-promoting environment, which may result in promotion of tumor growth and limitation of anti-neoplastic efficacy in some tumors. After paclitaxel and doxorubicin treatment in PyMT-MMTV mammary carcinoma, increased recruitment of TAMs was found to be mediated by increased CSF-1, CCL2 and CXCL2 [52, 171]. In murine Panc02 pancreatic cancer model, gemcitabine could induce Th2 cytokines from cancer cells to promote M2 polarized TAMs [10]. In a k-ras mutated murine pancreatic cancer model, gemcitabine induced recruitment of immature myeloid cells by GM-CSF secreted from damaged cancer cells which dampened the chemotherapeutic effects [172]. In vitro study, cisplatin and carboplatin increased the expression of IL-6 in 10 gynecologic malignant cancer cell lines to induce M2 polarized TAMs [18]. TAMs can limit the effects of chemotherapy or radiotherapy by various mechanisms, such as inhibition of cytotoxic T cells, activation of Th17 cells by inflammasome-IL1β, secreting of cathepsin, protection of cancer stem cells and alter vascular permeability to inhibit intratumoral drug concentration [172, 173]. Gemcitabine and 5-FU could also trigger cathepsin B release in MDSCs to activate the Nlrp3 inflammasome and promote tumor growth [172].

In contrary, some chemotherapeutic agents could also foster anti-tumor immunities. Doxorubicin could cause ICD in immunogenic tumor models to activate macrophages and dendritic cells to promote T cell response [174]. Doxorubicin also stimulated cancer cells to release ATP, which could recruit myeloid cells and induce differentiation into antigen presenting cells, finally resulting in effective antitumor immunities [175]. After cyclophosphamide treatment, leukemic cells released CCL4, CXCL8 and VEGF to recruit and active monocytes and enhance their phagocytic activity [176]. In murine EL4 lymphoma model, gemcitabine and 5-fluorouracil (5FU) were selectively cytotoxic on MDSCs and the elimination of MDSCs increased the toxicity of CD8(+) cells [177]. Docetaxel could deplete M2 polarized TAMs and activate M1 in 4T1-Neu mammary tumor implants [178]. Trabectedin inhibited the growth of murine fibrosarcomas partially by depletion of TAMs [179].

### Targeted therapies

Due to the discoveries of the molecular mechanisms of some malignancies, targeted therapies have been available to treat some tumors. Imatinib was primarily designed to treat Philadelphia chromosome positive chronic myeloid leukemia, and later it showed dramatical effects on gastrointestinal stromal tumors (GIST). In a murine GIST model, imatinib caused reduction of TAMs through CSF1R-CSF1 inhibition, however, converted TAMs to be M2 polarized type through C/EBPβ [180]. Sorafenib is a multi kinase inhibitor, including VEGFR2, and it showed active roles to treat hepatocellular carcinoma (HCC). In HCC xenograft murine model, sorafenib induced infiltration of TAMs via CXCL12, and depletion of TAMs potentiated the effects of sorafenib on angiogenesis, growth and metastasis of the tumor [181]. However, in another murine model of HCC, sorafinib was found to induce M1 polarized TAMs and to promote their stimulatory activities on NK cells [182]. Blockade of kit also showed abilities to inhibit the expansion of MDSCs and restore the immunity of T cells against tumors [183]. Antiangiogenic therapies based on inhibition of VEGF pathway could induce transient responses of tumors, however destruction of the angiogenesis created a strongly hypoxic microenvironment, which could recruit and activate MDSCs and TAMs and then they produce varieties of proangiogenic factors to stimulate angiogenesis [184]. In preclinical study, depletion of TAMs, either by clodronate-loaded liposomes or CSF-1R inhibition, increased the antitumor effects of VEGF-targeted therapies and as well combination anti-angiopoietin-2 with low-dose metronomic chemotherapy successfully inhibited the repopulation of myeloid cells and achieved synergic effects [185].

### Antibody based chemotherapy

Monoantibody based target therapies have shown promising effects for some kind of tumors. TAMs express Fc receptors that bind the Fc fragment of antibodies, engaging in Ab-dependent cellular cytotoxicity/phagocytosis (ADCC/ADCP). Trastuzumab, a moAb against the human epidermal growth factor receptor-2 (HER2), on one hand, directly inhibited HER2 signal pathway, on the other hand, induced ADCC and ADCP and primed CD8(+) T cell responses in breast cancer [186]. TAMs also enhance B cell lymphoma elimination in response to rituximab (a moAb against CD20) through FcgR-dependent ADCP and high infiltrations of TAMs were correlated with a better prognosis in rituximab treated patients. Immune checkpoints play vital roles to regulate the functions of T cells in tumor tissue. Molecules involved in checkpoint regulation include CTLA-4 and PDL1/PDL2 and TAMs express these immune checkpoint molecules. Recent evidence suggests that anti-CTLA-4 antibodies act via TAMs [187]. In murine models, depletion of Treg cells by macrophage-mediated ADCC was an essential component of the effects of anti-CTLA-4 [188]. However, it have been also reported that cetuximab, a moAb against EGFR was shown to enhance the immunosuppressive, proangiogenic, and protumoral functions of TAMs both in experimental tumor models and human cancers [189].

Taken together, the above studies showed the dual roles of chemotherapeutic drugs in regulating the tumor microenvironment which could significantly affect the efficacy of the treatments. The type of drugs, the sensitivities to the drugs of cancer cells, the immunogenic nature of cancer cells, the context of primary tumor microenvironment and the dynamic period after treatment should be considered to further delineate these interactions.

## Targeting tumor microenvironment of pancreatic cancer

The growing importance of the stromal cells in regulation of almost every aspect of tumor progression leads to the option of therapeutic applications of targeting these cells. These stromal cell-targeting therapies include inhibition of expansion, blockade of recruitment, inhibition of activation, induction of differentiation or repolarization to a tumor-suppression phenotype, and even just complete depletion of these cells (Additional file 1: Table S3).

### MDSCs and TAMs

The GM-CSF, G-CSF and CSF1 are key factors to promote proliferation and mobilize MDSCs and monocytes from bone marrow. Neutralizing antibodies to GM-CSF, G-CSF and CSF-1 have shown abilities to inhibit tumor growth in mice, including pancreatic cancer, colon cancer and lung cancer, by inhibition of proliferations of MDSCs and TAMs [52, 80, 190, 191]. Antibody to IL-6R [192], enzyme inhibitors, such as amino-bisphosphonate [193], PDE5 inhibitors [194], could inhibit proliferation of MDSCs to reduce the progression of breast cancer, colon cancer, fibrosarcoma in mice. Antibody or depletion of CCL2 blocked the recruitment of MDSCs and TAMs in tumor microenvironment and showed effects to inhibit pulmonary metastasis of murine mammary cancer [195]. As well, antagonists of CXCR2 and CXCR4 altered recruitment of MDSC to the tumor to inhibit metastasis of murine breast cancer [196]. Depletion of pan-TAMs by liposome-clodronate also showed abilities to inhibit tumor growth in various murine tumor models (e.g., teratocarcinoma, lung cancer, and melanoma) and human xenograft tumor models (e.g., cervical cancer, head and neck cancers) [46]. However, the obvious limitation of such treatment is the lack of specificity in depletion of different types of TAMs. In a murine squamous cell carcinogenesis model, repolarization of TAMs was more effective than blocking recruitment or depletion of TAMs, since macrophages are necessary for recruitment and activation of T cells under some circumstances [197]. Th2 type cytokines and COX-2 are main factors to induce MDSC and M2 polarized TAMs. Anti-IL-10 in addition with an inflammatory agent like CpG results in the transition of TAMs from M2 to M1 phenotype, resulted in tumor inhibitions. Aspirin and Celebrex, COX-2 inhibitors, showed ability to inhibit MDSCs and M2 to prevent pancreatic cancerogenesis and improve the effects of gemcitabine [10]. Th1 type cytokines are main inducers of M1 polarized TAMs. IL-12 treatment could re-program TAMs from M2 to M1 to increase anti-tumor response and tumor regression in a murine lung cancer model [198]. Since macrophages can be activated by Fc receptor of immunoglobulin, monoclonal antibody to HER2, CD20 and CD47 have showed to activate TAMs to enhance antitumor activities in murine breast cancer or non-hodgkin’s lymphoma [199, 200]. Increase of PD1 expression in TAMs and MDSCs has been found, anti-PD1 antibody also showed to activate TAMs and MDSCs in murine pancreatic cancer model [201]. CD40 agonist showed significant roles to activate the tumor-suppression effects of TAMs to improve the efficacy of gemcitabine in both murine pancreatic cancer model and early clinical trials [202]. All-trans retinoic acid (ATRA) and vitamin D could induce MDSC to differentiate into osteoclasts, and dendritic cells which reduce the immunosuppression [203]. Considering the ability of intratumoral infiltration of TAMs, TAMs also have been attempted to use as vehicles of drug delivery or other therapeutic interventions. Genetic modified TAMs expressing IFN-γ could induce antitumor and anti-angiogenic effects in murine tumor models [204]. TAM delivery of oncolytic virus showed to limit tumor re-growth following chemotherapy in a human prostate cancer xenograft model [205].

The strategies to target macrophages have shown promising effects, however the question remains which of these methods are more efficacious when combined with cytotoxic, targeted or immune checkpoint blockade therapy. Considering the potential anti-tumor effects of macrophages, reprogramming could be a better option than pan-macrophages inhibition, depletion or blockade of recruitment.

### PSCs and CAFs

According to the roles of CAFs and PSCs in pancreatic cancer, one would assume that CAFs and PSCs targeting may serve as powerful weapons to fight against pancreatic cancer and to improve therapeutic effects, however the up to date results are conflicting and more complicated than we can imagine. Sonic hedgehog (shh) pathway inhibitor IPI-926 was applied to deplete desmoplastic stroma and CAFs in pancreatic cancer, and the finding resulted in increased vascularization and more effective drug delivery of gemcitabine, with improved overall survival in KPC mouse model [206]. Clinical trials of anti-angiogenesis therapies did not show benefit in pancreatic cancer, when combined with gemcitabine [207, 208]. This finding could explain why these anti-angiogenesis therapies failed to improve the effects of gemcitabine, as these approaches would potentially lead to decrease intratumoral concentration of chemotherapeutic agents. However, when combined with the FOLFIRINOX regimen, IPI-926 led to a shorter median survival in pancreatic cancer patients [209]. The MMPs are the enzymes that are most responsible for degrading ECM components which potentially enhance the effects of gemcitabine. However, high expressions of MMP2, MMP7 and MMP11 in pancreatic cancer were found to be associated with a poor prognosis [210]. The clinical trials of MMP inhibitors, either alone or in combination with gemcitabine have not shown positive results [211]. Moreover, recent study based on PKT spontaneous pancreatic cancer mice model, depletion of desmoplastic stroma might promote the ability of cancer cells to invade the surrounding tissue and metastasize [212–214]. In accordance with results from PKT mice, small numbers of α-SMA positive CAFs in human pancreatic cancer tissue predicted shorter survival [212].

Vitamin D can induce quiescence of CAFs and aPSCs. Calcipotriol, a analogue of vitamin D, was administered with gemcitabine into KPC mice, resulting in obvious reduction of tumor in most of the mice, with a dramatical increase of intratumoral concentration of gemcitabine by 500% [215]. ATRA can also convert activated PSCs to quiescent PSCs to slow tumor progression and migration in mice pancreatic cancer model [216]. It is also believed that dense stroma tissue will increase interstitial fluid pressure (IFP) and then limits the delivery of chemotherapeutic drugs into cancer tissue. After treatment by PEGPH20, a hyaluronan-degrading enzyme, the IFP was decreased and functional perfusion of collapsed vascular structures was restored. Better survival was observed in pancreatic cancer bearing mice with a combination of PEPH20 and gemcitabine [217, 218]. Phase I clinical trial of PEGPH 20 showed no obvious toxicity and phase II clinical trial are planned [219]. Nab-paclitaxel is a combination of albumin and paclitaxel which has shown to improve the effects of gemcitabine. Albumin enables paclitaxel to transcytosis across endothelial cells through albumin receptors and then SPARC in tumor stroma has high affinity to albumin, which allows paclitaxel accumulation and then paclitaxel can induce stromal collapse, resulting greater efficacy of gemcitabine delivery and concentration in the tumor. In a current phase III clinical trial, the combination of nab-paclitaxel and gemcitabine have shown inspiring results [23]. In addition to the aspects of ECM, CAFs can also sequester T cells by expression of CXCL 12, and AMD3100, an inhibitor of CXCR4, could block the effects of CXCL12 of CAFs and enhanced the effects of antagonist of PDL1 in KPC mice [220].

Taken together, the above studies indicated the complicated effects of the desmoplastic tumor stroma targeting therapies. Most of the studies showed that depletion of desmoplasia, inactivation of PSCs and CAFs could improve the effects of gemcitabine in mice model, however the clinical trials did not get equal satisfactory results as in mice models. And even, recent studies supported the idea that the desmoplastic stroma might form a barrier that reduced the invasion and metastasis of cancer cells. Hence, the roles of desmoplastic stroma seem to be context-dependent during different stages of the tumor and under different treatment. Since the PSCs and fibroblasts have vital physiological roles, induction of quiescence of PSCs and CAFs, might be a better promising approach than complete ablation of desmoplastic stroma for future development of therapies targeting tumor desmoplasia of pancreatic cancer.

## Conclusion

**Fig. 1:**
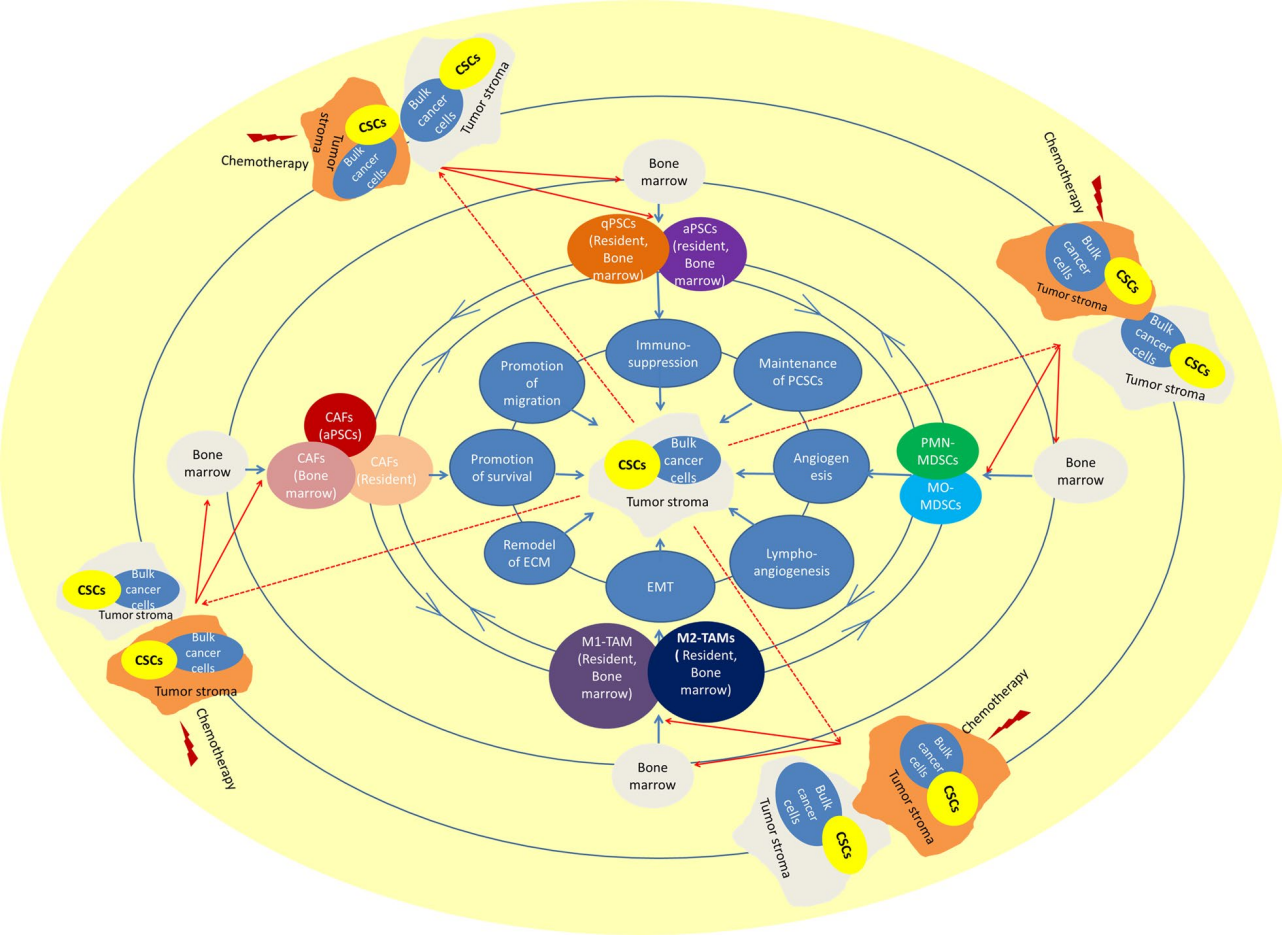
The landscape of tumor microenvironment (TME) of pancreatic cancer: (1) the PSCs, CAFs, MDSCs and TAMs promote the malignant biological behaviors of pancreatic cancer through eight aspects; (2) the phenotypes and functions of PSCs, CAFs, MDSCs and TMAs in TME of pancreatic cancer are dynamically changed and they can regulate each other; (3) bone marrow is the most importance origination for TAMs and MDSCs, and as well the bone marrow contributes to the PSCs and CAFs; (4) the cancer cells, including bulk cells and cancer stem cells (CSCs) in tumor tissue, are the main triggers to induce the architecture of TME, after chemotherapy, the damaged cancer cells, apoptotic cancer cells or immunogenic death of cancer cells can secrete varieties of signals to act on the stroma cells in TEM or to expand, recruit and activate bone marrow derived cells to remodel the TME, eventually affecting the efficacy of treatments or even leading to drug resistance

Pancreatic cancer will be the second leading cancer death in USA in 2030. Although tremendous efforts have been put on the study of pancreatic cancer cells, the improvements of survival have been minimally limited. The complicated network consisting of PSCs, CAFs, TAMs, MDSCs and cancer cells play crucial roles in pancreatic cancerogenesis, tumor progression, metastasis and drug responses. In addition to direct toxicities to cancer cells, chemotherapy can also remodel the TME, affecting the efficacy, or even contributing to drug resistance (Fig. 1). New treatments, targeting the tumor microenvironment, are highly warranted, however there are still some aspects need further explorations: (1) since Th2 cytokines are main cytokines to activate or polarize PSCs, CAFs, TAMs and MDSCs, it is of great importance to uncover why pancreatic cancer cells express high level of the Th2 cytokines; (2) there are many crosstalk between these five cell populations, which could dwarf the effects of any single target therapy, so combinational treatment may provide better results; (3) since of the diversities of the functions of PSCs, CAFs, TAMs and MDSCs, which could potentially contribute to anti-tumor effects, the regulations of the functions of these cells could be more effective than that of complete depletion of all of these cells; (4) since these stromal cells can seldom kill or damage cancer cells directly, the combinations of stroma cell-targeting treatments with direct cancer cell-targeting treatments could warrant better results; (5) among these five cell populations, M2 polarized TAMs express exclusive surface markers (e.g., CD206, CD163) which are seldom expressed on other immune cells or any other tissues, and there are also abundant infiltration of M2 polarized TAM in pancreatic cancer tissue, in contrast, these cells are seldom found in the peripheral blood or any other part of normal tissue, so these M2 exclusive surface markers could be applied as targets for directional intratumoral drug delivery.

## Additional file

**Additional file 1: Table S1.** The chronological list of landmark events of chemotherapy in pancreatic cancer from 2000. **Table S2.** The pro-tumoral and anti-tumoral remodeling effects of chemotherapy on TME. **Table S3.** Tumor microenvironment targeting therapies of pancreatic cancer.

## Abbreviations

ATRA: all-trans retinoic acid
ARG1: arginase1
bFGF: basic fibroblast growth factor
CAFs: cancer associated fibroblasts
CSF-1: colony stimulating factor-1
COX-2: cyclooxygenase-2
CSCs: cancer stem cells
CTCs: circulating tumor cells
CTLA-4: cytotoxic T-lymphocyte-associated protein 4
CXCL: c-x-c motif ligand
CXCR: receptor of c-x-c motif ligand
DAMPs: damage associated molecular patterns
DMBA: dimethylbenzanthracene
ECM: extracellular matrix
EMT: epithelial mesenchymal transition
FAP: fibroblast activation protein
GIST: gastrointestinal stromal tumors
GM/M-CSF: granulocyte macrophage/macrophage colony stimulating factor
GFAP: glial fibrillary acid protein
HCC: hepatocellular carcinoma
HMGB1: high-mobility group box1 protein
ICD: immunogenic cell death
iNOS: inducible nitric oxide synthase
IL: interleukin
IFP: interstitial fluid pressure
iMCs: immature myeloid cells
IFN-γ: interferon-γ
MDSCs: myeloid derived suppressor cells
MMPs: metal matrix proteases
PanIN: pancreatic intraductal neoplasia
PDAC: pancreatic ductal adenocarcinoma
PD-1: programmed death 1
PGE2: prostaglandin E2
PSCs: pancreatic stellate cells
STAT: signal transducer and activator of transcription
PCSCs: pancreatic cancer stem cells
PDGF: platelet-derived growth factor
TME: tumor microenvironment
Treg: regulatory T cells
TAMs: tumor associated macrophages
Th2: T helper 2
TGFβ: transforming growth factor beta
TNFα: tumor necrosis factor alpha
VEGF: vascular endothelial growth factor

## References

[1] Miller KD, Siegel RL, Lin CC (2016). Cancer treatment and survivorship statistics, 2016. CA Cancer J Clin.

[2] Neoptolemos JP, Dunn JA, Stocken DD (2001). Adjuvant chemoradiotherapy and chemotherapy in resectable pancreatic cancer: a randomised controlled trial. Lancet.

[3] Neoptolemos JP, Stocken DD, Bassi C (2010). Adjuvant chemotherapy with fluorouracil plus folinic acid vs gemcitabine following pancreatic cancer resection: a randomized controlled trial. JAMA.

[4] Uesaka K, Boku N, Fukutomi A (2016). Adjuvant chemotherapy of S-1 versus gemcitabine for resected pancreatic cancer: a phase 3, open-label, randomised, non-inferiority trial (JASPAC 01). Lancet.

[5] Conroy T, Desseigne F, Ychou M (2011). FOLFIRINOX versus gemcitabine for metastatic pancreatic cancer. N Engl J Med.

[6] Ferrone CR, Marchegiani G, Hong TS (2015). Radiological and surgical implications of neoadjuvant treatment with FOLFIRINOX for locally advanced and borderline resectable pancreatic cancer. Ann Surg.

[7] Golcher H, Brunner TB, Witzigmann H (2015). Neoadjuvant chemoradiation therapy with gemcitabine/cisplatin and surgery versus immediate surgery in resectable pancreatic cancer: results of the first prospective randomized phase II trial. Strahlenther Onkol.

[8] Neesse A, Algul H, Tuveson DA, Gress TM (2015). Stromal biology and therapy in pancreatic cancer: a changing paradigm. Gut.

[9] Apte M, Pirola RC, Wilson JS (2015). Pancreatic stellate cell: physiologic role, role in fibrosis and cancer. Curr Opin Gastroenterol.

[10] Liu Q, Li Y, Niu Z (2016). Atorvastatin (Lipitor) attenuates the effects of aspirin on pancreatic cancerogenesis and the chemotherapeutic efficacy of gemcitabine on pancreatic cancer by promoting M2 polarized tumor associated macrophages. J Exp Clin Cancer Res.

[11] Duluc C, Moatassim-Billah S, Chalabi-Dchar M (2015). Pharmacological targeting of the protein synthesis mTOR/4E-BP1 pathway in cancer-associated fibroblasts abrogates pancreatic tumour chemoresistance. EMBO Mol Med.

[12] Feig C, Jones JO, Kraman M (2013). Targeting CXCL12 from FAP-expressing carcinoma-associated fibroblasts synergizes with anti-PD-L1 immunotherapy in pancreatic cancer. Proc Natl Acad Sci USA.

[13] Zheng D, Chen H, Bartee MY (2013). Myxomaviral anti-inflammatory serpin reduces myeloid-derived suppressor cells and human pancreatic cancer cell growth in mice. J Cancer Sci Ther.

[14] Yao L, Wang M, Niu Z (2016). Interleukin-27 inhibits malignant behaviors of pancreatic cancer cells by targeting M2 polarized tumor associated macrophages. Cytokine.

[15] Gabitass RF, Annels NE, Crawshaw J, Pandha HS, Middleton GW (2011). Use of gemcitabine-(Gem) and fluropyrimidine (FP)-based chemotherapy to reduce myeloid-derived suppressor cells (MDSCs) in pancreatic (PC) and esophagogastric cancer (EGC). J Clin Oncol..

[16] Liu QF, Li Y, Niu ZY (2016). Atorvastatin (Lipitor) attenuates the effects of aspirin on pancreatic cancerogenesis and the chemotherapeutic efficacy of gemcitabine on pancreatic cancer by promoting M2 polarized tumor associated macrophages. J Exp Clin Cancer Res.

[17] Takeuchi S, Baghdadi M, Tsuchikawa T (2015). Chemotherapy-derived inflammatory responses accelerate the formation of immunosuppressive myeloid cells in the tissue microenvironment of human pancreatic cancer. Cancer Res.

[18] Dijkgraaf EM, Heusinkveld M, Tummers B (2013). Chemotherapy alters monocyte differentiation to favor generation of cancer-supporting M2 macrophages in the tumor microenvironment. Cancer Res.

[19] Neoptolemos JP, Stocken DD, Friess H (2004). A randomized trial of chemoradiotherapy and chemotherapy after resection of pancreatic cancer. N Engl J Med.

[20] Oettle H, Post S, Neuhaus P (2007). Adjuvant chemotherapy with gemcitabine vs observation in patients undergoing curative-intent resection of pancreatic cancer—a randomized controlled trial. JAMA.

[21] Oettle H, Neuhaus P, Hochhaus A (2013). Adjuvant chemotherapy with gemcitabine and long-term outcomes among patients with resected pancreatic cancer the CONKO-001 randomized trial. JAMA.

[22] Neoptolemos JP, Palmer DH, Ghaneh P (2017). Comparison of adjuvant gemcitabine and capecitabine with gemcitabine monotherapy in patients with resected pancreatic cancer (ESPAC-4): a multicentre, open-label, randomised, phase 3 trial. Lancet.

[23] Von Hoff DD, Ervin T, Arena FP (2013). Increased survival in pancreatic cancer with nab-paclitaxel plus gemcitabine. N Engl J Med.

[24] Oettle H, Riess H, Stieler JM (2014). Second-line oxaliplatin, folinic acid, and fluorouracil versus folinic acid and fluorouracil alone for gemcitabine-refractory pancreatic cancer: outcomes from the CONKO-003 trial. J Clin Oncol.

[25] Wang-Gillam A, Li CP, Bodoky G (2016). Nanoliposomal irinotecan with fluorouracil and folinic acid in metastatic pancreatic cancer after previous gemcitabine-based therapy (NAPOLI-1): a global, randomised, open-label, phase 3 trial. Lancet.

[26] Gillen S, Schuster T, Zum Buschenfelde CM, Friess H, Kleeff J (2010). Preoperative/neoadjuvant therapy in pancreatic cancer: a systematic review and meta-analysis of response and resection percentages. PLoS Med.

[27] Mews P, Phillips P, Fahmy R (2002). Pancreatic stellate cells respond to inflammatory cytokines: potential role in chronic pancreatitis. Gut.

[28] Bhatia V, Rastellini C, Han S (2014). Acinar cell-specific knockout of the PTHrP gene decreases the proinflammatory and profibrotic responses in pancreatitis. Am J Physiol Gastrointest Liver Physiol.

[29] Pandol S, Gukovskaya A, Edderkaoui M (2012). Epidemiology, risk factors, and the promotion of pancreatic cancer: role of the stellate cell. J Gastroenterol Hepatol.

[30] Fukushima N, Kikuchi Y, Nishiyama T, Kudo A, Fukayama M (2008). Periostin deposition in the stroma of invasive and intraductal neoplasms of the pancreas. Mod Pathol.

[31] Ikenaga N, Ohuchida K, Mizumoto K (2010). CD10+ pancreatic stellate cells enhance the progression of pancreatic cancer. Gastroenterology..

[32] Fujiwara K, Ohuchida K, Mizumoto K (2012). CD271(+) subpopulation of pancreatic stellate cells correlates with prognosis of pancreatic cancer and is regulated by interaction with cancer cells. PLoS ONE.

[33] Sugimoto H, Mundel TM, Kieran MW, Kalluri R (2006). Identification of fibroblast heterogeneity in the tumor microenvironment. Cancer Biol Ther.

[34] Polanska UM, Orimo A (2013). Carcinoma-associated fibroblasts: non-neoplastic tumour-promoting mesenchymal cells. J Cell Physiol.

[35] Kawase A, Ishii G, Nagai K (2008). Podoplanin expression by cancer associated fibroblasts predicts poor prognosis of lung adenocarcinoma. Int J Cancer.

[36] Nielsen MF, Mortensen MB, Detlefsen S (2016). Key players in pancreatic cancer-stroma interaction: cancer-associated fibroblasts, endothelial and inflammatory cells. World J Gastroenterol.

[37] Erez N, Truitt M, Olson P, Hanahan D (2010). Cancer-associated fibroblasts are activated in incipient neoplasia to orchestrate tumor-promoting inflammation in an NF-kappa B-dependent manner. Cancer Cell.

[38] Rasanen K, Vaheri A (2010). Activation of fibroblasts in cancer stroma. Exp Cell Res.

[39] Hoshino A, Ishii G, Ito T (2011). Podoplanin-positive fibroblasts enhance lung adenocarcinoma tumor formation: podoplanin in fibroblast functions for tumor progression. Cancer Res.

[40] Wikberg ML, Edin S, Lundberg IV (2013). High intratumoral expression of fibroblast activation protein (FAP) in colon cancer is associated with poorer patient prognosis. Tumour Biol.

[41] Zhao H, Peehl DM (2009). Tumor-promoting phenotype of CD90hi prostate cancer-associated fibroblasts. Prostate.

[42] Tang Y, Katuri V, Srinivasan R (2005). Transforming growth factor-beta suppresses nonmetastatic colon cancer through Smad4 and adaptor protein ELF at an early stage of tumorigenesis. Cancer Res.

[43] Flaberg E, Markasz L, Petranyi G (2011). High-throughput live-cell imaging reveals differential inhibition of tumor cell proliferation by human fibroblasts. Int J Cancer.

[44] Takahashi A, Ishii G, Neri S (2015). Podoplanin-expressing cancer-associated fibroblasts inhibit small cell lung cancer growth. Oncotarget.

[45] Clark CE, Hingorani SR, Mick R (2007). Dynamics of the immune reaction to pancreatic cancer from inception to invasion. Cancer Res.

[46] Biswas SK, Allavena P, Mantovani A (2013). Tumor-associated macrophages: functional diversity, clinical significance, and open questions. Semin Immunopathol.

[47] Yona S, Kim KW, Wolf Y (2013). Fate mapping reveals origins and dynamics of monocytes and tissue macrophages under homeostasis. Immunity.

[48] Ginhoux F, Greter M, Leboeuf M (2010). Fate mapping analysis reveals that adult microglia derive from primitive macrophages. Science.

[49] Wynn TA, Chawla A, Pollard JW (2013). Macrophage biology in development, homeostasis and disease. Nature.

[50] Hamilton JA, Achuthan A (2013). Colony stimulating factors and myeloid cell biology in health and disease. Trends Immunol.

[51] Quail DF, Joyce JA (2013). Microenvironmental regulation of tumor progression and metastasis. Nat Med.

[52] DeNardo DG, Brennan DJ, Rexhepaj E (2011). Leukocyte complexity predicts breast cancer survival and functionally regulates response to chemotherapy. Cancer Discov.

[53] Linde N, Lederle W, Depner S (2012). Vascular endothelial growth factor-induced skin carcinogenesis depends on recruitment and alternative activation of macrophages. J Pathol.

[54] Franklin RA, Liao W, Sarkar A (2014). The cellular and molecular origin of tumor-associated macrophages. Science.

[55] Su S, Liu Q, Chen J (2014). A positive feedback loop between mesenchymal-like cancer cells and macrophages is essential to breast cancer metastasis. Cancer Cell.

[56] Kitamura T, Fujishita T, Loetscher P (2010). Inactivation of chemokine (CC motif) receptor 1 (CCR1) suppresses colon cancer liver metastasis by blocking accumulation of immature myeloid cells in a mouse model. Proc Natl Acad Sci USA.

[57] Cortez-Retamozo V, Etzrodt M, Newton A (2013). Angiotensin II drives the production of tumor-promoting macrophages. Immunity.

[58] Okabe Y, Medzhitov R (2014). Tissue-specific signals control reversible program of localization and functional polarization of macrophages. Cell.

[59] Colegio OR, Chu NQ, Szabo AL (2014). Functional polarization of tumour-associated macrophages by tumour-derived lactic acid. Nature.

[60] Satoh T, Kidoya H, Naito H (2013). Critical role of Trib1 in differentiation of tissue-resident M2-like macrophages. Nature.

[61] Stein M, Keshav S, Harris N, Gordon S (1992). Interleukin 4 potently enhances murine macrophage mannose receptor activity: a marker of alternative immunologic macrophage activation. J Exp Med.

[62] Mills CD, Kincaid K, Alt JM, Heilman MJ, Hill AM (2000). M-1/M-2 macrophages and the Th1/Th2 paradigm. J Immunol.

[63] Biswas SK, Mantovani A (2010). Macrophage plasticity and interaction with lymphocyte subsets: cancer as a paradigm. Nat Immunol.

[64] Murray PJ, Allen JE, Biswas SK (2014). Macrophage activation and polarization: nomenclature and experimental guidelines. Immunity.

[65] Mantovani A, Allavena P (2015). The interaction of anticancer therapies with tumor-associated macrophages. J Exp Med.

[66] Leblond MM, Gerault AN, Corroyer-Dulmont A (2016). Hypoxia induces macrophage polarization and re-education toward an M2 phenotype in U87 and U251 glioblastoma models. Oncoimmunology.

[67] Guo X, Xue H, Shao Q (2016). Hypoxia promotes glioma-associated macrophage infiltration via periostin and subsequent M2 polarization by upregulating TGF-beta and M-CSFR. Oncotarget.

[68] Colegio OR (2016). Lactic acid polarizes macrophages to a tumor-promoting state. Oncoimmunology.

[69] Prima V, Kaliberova LN, Kaliberov S, Curiel DT, Kusmartsev S (2017). COX2/mPGES1/PGE2 pathway regulates PD-L1 expression in tumor-associated macrophages and myeloid-derived suppressor cells. Proc Natl Acad Sci USA.

[70] Rojas A, Delgado-Lopez F, Perez-Castro R (2016). HMGB1 enhances the protumoral activities of M2 macrophages by a RAGE-dependent mechanism. Tumour Biol.

[71] Ruffell B, Coussens LM (2015). Macrophages and therapeutic resistance in cancer. Cancer Cell.

[72] Talmadge JE, Gabrilovich DI (2013). History of myeloid-derived suppressor cells. Nat Rev Cancer.

[73] Lappat EJ, Cawein M (1964). A study of the leukemoid response to transplantable A-280 tumor in mice. Cancer Res.

[74] Gabrilovich DI, Bronte V, Chen SH (2007). The terminology issue for myeloid-derived suppressor cells. Cancer Res.

[75] Youn JI, Nagaraj S, Collazo M, Gabrilovich DI (2008). Subsets of myeloid-derived suppressor cells in tumor-bearing mice. J Immunol.

[76] Movahedi K, Guilliams M, Van den Bossche J (2008). Identification of discrete tumor-induced myeloid-derived suppressor cell subpopulations with distinct T cell-suppressive activity. Blood.

[77] Peranzoni E, Zilio S, Marigo I (2010). Myeloid-derived suppressor cell heterogeneity and subset definition. Curr Opin Immunol.

[78] Solito S, Marigo I, Pinton L (2014). Myeloid-derived suppressor cell heterogeneity in human cancers. Year Immunol Myeloid Cells Inflamm.

[79] Condamine T, Gabrilovich DI (2011). Molecular mechanisms regulating myeloid-derived suppressor cell differentiation and function. Trends Immunol.

[80] Bayne LJ, Beatty GL, Jhala N (2012). Tumor-derived granulocyte-macrophage colony-stimulating factor regulates myeloid inflammation and T cell immunity in pancreatic cancer. Cancer Cell.

[81] Marvel D, Gabrilovich DI (2015). Myeloid-derived suppressor cells in the tumor microenvironment: expect the unexpected. J Clin Investig.

[82] Fang Z, Li J, Yu X (2015). Polarization of monocytic myeloid-derived suppressor cells by hepatitis B surface antigen is mediated via ERK/IL-6/STAT3 signaling feedback and restrains the activation of T cells in chronic hepatitis B virus infection. J Immunol.

[83] Chen MF, Kuan FC, Yen TC (2014). IL-6-stimulated CD11b+ CD14+ HLA-DR—myeloid-derived suppressor cells, are associated with progression and poor prognosis in squamous cell carcinoma of the esophagus. Oncotarget.

[84] Lee CR, Kwak Y, Yang T (2016). Myeloid-derived suppressor cells are controlled by regulatory T cells via TGF-beta during murine colitis. Cell Rep.

[85] Gabrilovich DI, Nagaraj S (2009). Myeloid-derived suppressor cells as regulators of the immune system. Nat Rev Immunol.

[86] Bronte V, Serafini P, De Santo C (2003). IL-4-induced arginase 1 suppresses alloreactive T cells in tumor-bearing mice. J Immunol.

[87] Ko JS (2010). Direct and differential suppression of myeloid-derived suppressor cell subsets by sunitinib is compartmentally constrained (vol 70, pg 3526, 2010). Cancer Res.

[88] Vasquez-Dunddel D, Pan F, Zeng Q (2013). STAT3 regulates arginase-I in myeloid-derived suppressor cells from cancer patients. J Clin Investig.

[89] Marigo I, Bosio E, Solito S (2010). Tumor-induced tolerance and immune suppression depend on the C/EBP beta transcription factor. Immunity.

[90] Condamine T, Ramachandran I, Youn JI, Gabrilovich DI (2015). Regulation of tumor metastasis by myeloid-derived suppressor cells. Annu Rev Med.

[91] Youn JI, Nagaraj S, Collazo M, Gabrilovich DI (2008). Subsets of myeloid-derived suppressor cells in tumor-bearing mice. J Immunol.

[92] Nagaraj S, Gupta K, Pisarev V (2007). Altered recognition of antigen is a mechanism of CD8(+) T cell tolerance in cancer. Nat Med.

[93] Nakamura T, Nakao T, Ashihara E, Yoshimura N (2016). Myeloid-derived suppressor cells recruit CD4(+)/Foxp3(+) regulatory T cells in a murine cardiac allograft. Transpl Proc.

[94] Kang X, Zhang X, Liu Z (2016). Granulocytic myeloid-derived suppressor cells maintain feto-maternal tolerance by inducing Foxp3 expression in CD4+ CD25-T cells by activation of the TGF-beta/beta-catenin pathway. Mol Hum Reprod.

[95] Sorrentino C, Miele L, Porta A, Pinto A, Morello S (2015). Myeloid-derived suppressor cells contribute to A2B adenosine receptor-induced VEGF production and angiogenesis in a mouse melanoma model. Oncotarget.

[96] Yang L, DeBusk LM, Fukuda K (2004). Expansion of myeloid immune suppressor Gr+ CD11b+ cells in tumor-bearing host directly promotes tumor angiogenesis. Cancer Cell.

[97] Wang J, Su X, Yang L (2016). The influence of myeloid-derived suppressor cells on angiogenesis and tumor growth after cancer surgery. Int J Cancer.

[98] Shojaei F, Wu X, Zhong C (2007). Bv8 regulates myeloid-cell-dependent tumour angiogenesis. Nature.

[99] Cui TX, Kryczek I, Zhao L (2013). Myeloid-derived suppressor cells enhance stemness of cancer cells by inducing microRNA101 and suppressing the corepressor CtBP2. Immunity.

[100] Liu Q, Liao Q, Zhao Y (2016). Myeloid-derived suppressor cells (MDSC) facilitate distant metastasis of malignancies by shielding circulating tumor cells (CTC) from immune surveillance. Med Hypotheses.

[101] Gabitass RF, Annels NE, Stocken DD, Pandha HA, Middleton GW (2011). Elevated myeloid-derived suppressor cells in pancreatic, esophageal and gastric cancer are an independent prognostic factor and are associated with significant elevation of the Th2 cytokine interleukin-13. Cancer Immunol Immunother.

[102] Nagathihalli NS, Castellanos JA, VanSaun MN (2016). Pancreatic stellate cell secreted IL-6 stimulates STAT3 dependent invasiveness of pancreatic intraepithelial neoplasia and cancer cells. Oncotarget.

[103] Tang D, Wang D, Yuan Z (2013). Persistent activation of pancreatic stellate cells creates a microenvironment favorable for the malignant behavior of pancreatic ductal adenocarcinoma. Int J Cancer.

[104] Lonardo E, Frias-Aldeguer J, Hermann PC, Heeschen C (2012). Pancreatic stellate cells form a niche for cancer stem cells and promote their self-renewal and invasiveness. Cell Cycle.

[105] Hamada S, Masamune A, Takikawa T (2012). Pancreatic stellate cells enhance stem cell-like phenotypes in pancreatic cancer cells. Biochem Biophys Res Commun.

[106] Sada M, Ohuchida K, Horioka K (2016). Hypoxic stellate cells of pancreatic cancer stroma regulate extracellular matrix fiber organization and cancer cell motility. Cancer Lett.

[107] Lu J, Zhou S, Siech M (2014). Pancreatic stellate cells promote hapto-migration of cancer cells through collagen I-mediated signalling pathway. Br J Cancer.

[108] Ikenaga N, Ohuchida K, Mizumoto K (2010). CD10(+) pancreatic stellate cells enhance the progression of pancreatic cancer. Gastroenterology.

[109] Kang Y, Roife D, Lee Y (2016). Transforming growth factor-beta limits secretion of lumican by activated stellate cells within primary pancreatic adenocarcinoma tumors. Clin Cancer Res.

[110] Drifka CR, Loeffler AG, Esquibel CR (2016). Human pancreatic stellate cells modulate 3D collagen alignment to promote the migration of pancreatic ductal adenocarcinoma cells. Biomed Microdevices.

[111] Wu YS, Looi CY, Subramaniam KS, Masamune A, Chung I (2016). Soluble factors from stellate cells induce pancreatic cancer cell proliferation via Nrf2-activated metabolic reprogramming and ROS detoxification. Oncotarget.

[112] Sousa CM, Biancur DE, Wang X (2016). Pancreatic stellate cells support tumour metabolism through autophagic alanine secretion. Nature..

[113] Mantoni TS, Lunardi S, Al-Assar O, Masamune A, Brunner TB (2011). Pancreatic stellate cells radioprotect pancreatic cancer cells through beta 1-integrin signaling. Cancer Res.

[114] Yoshida N, Masamune A, Hamada S (2017). Kindlin-2 in pancreatic stellate cells promotes the progression of pancreatic cancer. Cancer Lett.

[115] Endo S, Nakata K, Ohuchida K (2017). Autophagy is required for activation of pancreatic stellate cells, associated with pancreatic cancer progression and promotes growth of pancreatic tumors in mice. Gastroenterology.

[116] Kikuta K, Masamune A, Watanabe T (2010). Pancreatic stellate cells promote epithelial–mesenchymal transition in pancreatic cancer cells. Biochem Biophys Res Commun.

[117] Gao Z, Wang X, Wu K, Zhao Y, Hu G (2010). Pancreatic stellate cells increase the invasion of human pancreatic cancer cells through the stromal cell-derived factor-1/CXCR4 axis. Pancreatology.

[118] Takikawa T, Masamune A, Yoshida N (2017). Exosomes derived from pancreatic stellate cells: microrna signature and effects on pancreatic cancer cells. Pancreas.

[119] Rybicka A, Eyileten C, Taciak B (2016). Tumour-associated macrophages influence canine mammary cancer stem-like cells enhancing their pro-angiogenic properties. J Physiol Pharmacol.

[120] Ali S, Suresh R, Banerjee S (2015). Contribution of microRNAs in understanding the pancreatic tumor microenvironment involving cancer associated stellate and fibroblast cells. Am J Cancer Res.

[121] Lunardi S, Muschel RJ, Brunner TB (2014). The stromal compartments in pancreatic cancer: are there any therapeutic targets?. Cancer Lett.

[122] Lee HO, Mullins SR, Franco-Barraza J (2011). FAP-overexpressing fibroblasts produce an extracellular matrix that enhances invasive velocity and directionality of pancreatic cancer cells. BMC Cancer.

[123] Arnold SA, Rivera LB, Miller AF (2010). Lack of host SPARC enhances vascular function and tumor spread in an orthotopic murine model of pancreatic carcinoma. Dis Model Mech.

[124] Kawase T, Yasui Y, Nishina S (2015). Fibroblast activation protein-alpha-expressing fibroblasts promote the progression of pancreatic ductal adenocarcinoma. BMC Gastroenterol.

[125] Xiao Q, Zhou D, Rucki AA (2016). Cancer-associated fibroblasts in pancreatic cancer are reprogrammed by tumor-induced alterations in genomic DNA methylation. Cancer Res.

[126] Roy I, Zimmerman NP, Mackinnon AC (2014). CXCL12 chemokine expression suppresses human pancreatic cancer growth and metastasis. PLoS ONE.

[127] Suklabaidya S, Dash P, Senapati S (2017). Pancreatic fibroblast exosomes regulate survival of cancer cells. Oncogene.

[128] Hirakawa T, Yashiro M, Doi Y (2016). Pancreatic fibroblasts stimulate the motility of pancreatic cancer cells through IGF1/IGF1R signaling under hypoxia. PLoS ONE.

[129] Leca J, Martinez S, Lac S (2016). Cancer-associated fibroblast-derived annexin A6+ extracellular vesicles support pancreatic cancer aggressiveness. J Clin Investig.

[130] Guan J, Zhang H, Wen Z (2014). Retinoic acid inhibits pancreatic cancer cell migration and EMT through the downregulation of IL-6 in cancer associated fibroblast cells. Cancer Lett.

[131] Zheng B, Ohuchida K, Chijiiwa Y (2016). CD146 attenuation in cancer-associated fibroblasts promotes pancreatic cancer progression. Mol Carcinog.

[132] Yang J, Liao D, Chen C (2013). Tumor-associated macrophages regulate murine breast cancer stem cells through a novel paracrine EGFR/Stat3/Sox-2 signaling pathway. Stem Cells.

[133] Sainz B, Carron E, Vallespinos M, Machado HL (2016). Cancer stem cells and macrophages: implications in tumor biology and therapeutic strategies. Mediators Inflamm.

[134] Li N, Li Y, Li Z (2016). Hypoxia inducible factor 1 (HIF-1) recruits macrophage to activate pancreatic stellate cells in pancreatic ductal adenocarcinoma. Int J Mol Sci.

[135] Nielsen SR, Quaranta V, Linford A (2016). Macrophage-secreted granulin supports pancreatic cancer metastasis by inducing liver fibrosis. Nat Cell Biol.

[136] Shi C, Washington MK, Chaturvedi R (2014). Fibrogenesis in pancreatic cancer is a dynamic process regulated by macrophage-stellate cell interaction. Lab Investig.

[137] Weizman N, Krelin Y, Shabtay-Orbach A (2014). Macrophages mediate gemcitabine resistance of pancreatic adenocarcinoma by upregulating cytidine deaminase. Oncogene.

[138] Gironella M, Calvo C, Fernandez A (2013). Reg3beta deficiency impairs pancreatic tumor growth by skewing macrophage polarization. Cancer Res.

[139] Cavel O, Shomron O, Shabtay A (2012). Endoneurial macrophages induce perineural invasion of pancreatic cancer cells by secretion of GDNF and activation of RET tyrosine kinase receptor. Cancer Res.

[140] Neyen C, Pluddemann A, Mukhopadhyay S (2013). Macrophage scavenger receptor a promotes tumor progression in murine models of ovarian and pancreatic cancer. J Immunol.

[141] Hermano E, Meirovitz A, Meir K, et al. Macrophage polarization in pancreatic carcinoma: role of heparanase enzyme. J Natl Cancer Inst. 2014;106(12):dju332. 10.1093/jnci/dju332 PMC4334800 25326645

[142] Panni RZ, Sanford DE, Belt BA (2014). Tumor-induced STAT3 activation in monocytic myeloid-derived suppressor cells enhances stemness and mesenchymal properties in human pancreatic cancer. Cancer Immunol Immunother.

[143] Stromnes IM, Brockenbrough JS, Izeradjene K (2014). Targeted depletion of an MDSC subset unmasks pancreatic ductal adenocarcinoma to adaptive immunity. Gut.

[144] Karnevi E, Rosendahl AH, Hilmersson KS, Saleem MA, Andersson R (2016). Impact by pancreatic stellate cells on epithelial-mesenchymal transition and pancreatic cancer cell invasion: adding a third dimension in vitro. Exp Cell Res.

[145] Wu YS, Chung I, Wong WF (2017). Paracrine IL-6 signaling mediates the effects of pancreatic stellate cells on epithelial-mesenchymal transition via Stat3/Nrf2 pathway in pancreatic cancer cells. Biochim Biophys Acta.

[146] Xu Z, Vonlaufen A, Phillips PA (2010). Role of pancreatic stellate cells in pancreatic cancer metastasis. Am J Pathol.

[147] Patel MB, Pothula SP, Xu Z (2014). The role of the hepatocyte growth factor/c-MET pathway in pancreatic stellate cell-endothelial cell interactions: antiangiogenic implications in pancreatic cancer. Carcinogenesis.

[148] Masamune A, Kikuta K, Watanabe T (2008). Hypoxia stimulates pancreatic stellate cells to induce fibrosis and angiogenesis in pancreatic cancer. Am J Physiol Gastrointest Liver Physiol.

[149] Tang D, Gao J, Wang S (2015). Apoptosis and anergy of T cell induced by pancreatic stellate cells-derived galectin-1 in pancreatic cancer. Tumor Biol.

[150] Tang D, Yuan Z, Xue X (2012). High expression of Galectin-1 in pancreatic stellate cells plays a role in the development and maintenance of an immunosuppressive microenvironment in pancreatic cancer. Int J Cancer.

[151] Mace TA, Bloomston M, Lesinski GB (2013). Pancreatic cancer-associated stellate cells a viable target for reducing immunosuppression in the tumor microenvironment. Oncoimmunology.

[152] Mace TA, Ameen Z, Collins A (2013). Pancreatic cancer-associated stellate cells promote differentiation of myeloid-derived suppressor cells in a STAT3-dependent manner. Cancer Res.

[153] Ene-Obong A, Clear AJ, Watt J (2013). Activated pancreatic stellate cells sequester CD8(+) T cells to reduce their infiltration of the juxtatumoral compartment of pancreatic ductal adenocarcinoma. Gastroenterology.

[154] Ma Y, Hwang RF, Logsdon CD, Ullrich SE (2013). Dynamic mast cell-stromal cell interactions promote growth of pancreatic cancer. Cancer Res.

[155] Li D, Qu C, Ning Z (2016). Radiation promotes epithelial-to-mesenchymal transition and invasion of pancreatic cancer cell by activating carcinoma-associated fibroblasts. Am J Cancer Res.

[156] Moatassim-Billah S, Duluc C, Samain R (2016). Anti-metastatic potential of somatostatin analog SOM230: indirect pharmacological targeting of pancreatic cancer-associated fibroblasts. Oncotarget.

[157] Pan B, Liao Q, Niu Z, Zhou L, Zhao Y (2015). Cancer-associated fibroblasts in pancreatic adenocarcinoma. Future Oncol.

[158] Ino Y, Yamazaki-Itoh R, Oguro S (2013). Arginase II expressed in cancer-associated fibroblasts indicates tissue hypoxia and predicts poor outcome in patients with pancreatic cancer. PLoS ONE.

[159] Roda O, Ortiz-Zapater E, Martinez-Bosch N, et al. Galectin-1 is a novel functional receptor for tissue plasminogen activator in pancreatic cancer. Gastroenterology. 2009; 136(4):1379–90, e1371–75. 10.1053/j.gastro.2008.12.039 19171142

[160] Zhang A, Qian Y, Ye Z (2017). Cancer-associated fibroblasts promote M2 polarization of macrophages in pancreatic ductal adenocarcinoma. Cancer Med.

[161] Liu CY, Xu JY, Shi XY (2013). M2-polarized tumor-associated macrophages promoted epithelial–mesenchymal transition in pancreatic cancer cells, partially through TLR4/IL-10 signaling pathway. Lab Investig.

[162] Helm O, Held-Feindt J, Grage-Griebenow E (2014). Tumor-associated macrophages exhibit pro- and anti-inflammatory properties by which they impact on pancreatic tumorigenesis. Int J Cancer.

[163] Griesmann H, Drexel C, Milosevic N (2016). Pharmacological macrophage inhibition decreases metastasis formation in a genetic model of pancreatic cancer. Gut..

[164] Zhu Y, Knolhoff BL, Meyer MA (2014). CSF1/CSF1R blockade reprograms tumor-infiltrating macrophages and improves response to T-cell checkpoint immunotherapy in pancreatic cancer models. Cancer Res.

[165] Seifert L, Werba G, Tiwari S (2016). Radiation therapy induces macrophages to suppress T-cell responses against pancreatic tumors in mice. Gastroenterology.

[166] Beatty GL, Winograd R, Evans RA (2015). Exclusion of T cells from pancreatic carcinomas in mice is regulated by Ly6C(low) F4/80(+) extratumoral macrophages. Gastroenterology.

[167] Zhao F, Obermann S, von Wasielewski R (2009). Increase in frequency of myeloid-derived suppressor cells in mice with spontaneous pancreatic carcinoma. Immunology.

[168] Porembka MR, Mitchem JB, Belt BA (2012). Pancreatic adenocarcinoma induces bone marrow mobilization of myeloid-derived suppressor cells which promote primary tumor growth. Cancer Immunol Immunother.

[169] Song J, Lee J, Kim J (2016). Pancreatic adenocarcinoma up-regulated factor (PAUF) enhances the accumulation and functional activity of myeloid-derived suppressor cells (MDSCs) in pancreatic cancer. Oncotarget.

[170] Pilon-Thomas S, Nelson N, Vohra N (2011). Murine pancreatic adenocarcinoma dampens SHIP-1 expression and alters MDSC homeostasis and function. PLoS ONE.

[171] Nakasone ES, Askautrud HA, Kees T (2012). Imaging tumor-stroma interactions during chemotherapy reveals contributions of the microenvironment to resistance. Cancer Cell.

[172] Bruchard M, Mignot G, Derangere V (2013). Chemotherapy-triggered cathepsin B release in myeloid-derived suppressor cells activates the Nlrp3 inflammasome and promotes tumor growth. Nat Med.

[173] Shree T, Olson OC, Elie BT (2011). Macrophages and cathepsin proteases blunt chemotherapeutic response in breast cancer. Genes Dev.

[174] Kroemer G, Galluzzi L, Kepp O, Zitvogel L (2013). Immunogenic cell death in cancer therapy. Annu Rev Immunol.

[175] Ma Y, Galluzzi L, Zitvogel L, Kroemer G (2013). Autophagy and cellular immune responses. Immunity.

[176] Pallasch CP, Leskov I, Braun CJ (2014). Sensitizing protective tumor microenvironments to antibody-mediated therapy. Cell.

[177] Vincent J, Mignot G, Chalmin F (2010). 5-Fluorouracil selectively kills tumor-associated myeloid-derived suppressor cells resulting in enhanced T cell-dependent antitumor immunity. Cancer Res.

[178] Kodumudi KN, Woan K, Gilvary DL (2010). A novel chemoimmunomodulating property of docetaxel: suppression of myeloid-derived suppressor cells in tumor bearers. Clin Cancer Res.

[179] Germano G, Frapolli R, Belgiovine C (2013). Role of macrophage targeting in the antitumor activity of trabectedin. Cancer Cell.

[180] Cavnar MJ, Zeng S, Kim TS (2013). KIT oncogene inhibition drives intratumoral macrophage M2 polarization. J Exp Med.

[181] Zhang CC, Yan Z, Zhang Q (2010). PF-03732010: a fully human monoclonal antibody against P-cadherin with antitumor and antimetastatic activity. Clin Cancer Res.

[182] Sprinzl MF, Reisinger F, Puschnik A (2013). Sorafenib perpetuates cellular anticancer effector functions by modulating the crosstalk between macrophages and natural killer cells. Hepatology.

[183] Pan PY, Wang GX, Yin B (2008). Reversion of immune tolerance in advanced malignancy: modulation of myeloid-derived suppressor cell development by blockade of stem-cell factor function. Blood.

[184] Mazzieri R, Pucci F, Moi D (2011). Targeting the ANG2/TIE2 axis inhibits tumor growth and metastasis by impairing angiogenesis and disabling rebounds of proangiogenic myeloid cells. Cancer Cell.

[185] Srivastava K, Hu J, Korn C (2014). Postsurgical adjuvant tumor therapy by combining anti-angiopoietin-2 and metronomic chemotherapy limits metastatic growth. Cancer Cell.

[186] Sliwkowski MX, Mellman I (2013). Antibody therapeutics in cancer. Science.

[187] Selby MJ, Engelhardt JJ, Quigley M (2013). Anti-CTLA-4 antibodies of IgG2a isotype enhance antitumor activity through reduction of intratumoral regulatory T cells. Cancer Immunol Res.

[188] Simpson TR, Li F, Montalvo-Ortiz W (2013). Fc-dependent depletion of tumor-infiltrating regulatory T cells co-defines the efficacy of anti-CTLA-4 therapy against melanoma. J Exp Med.

[189] Pander J, Heusinkveld M, van der Straaten T (2011). Activation of tumor-promoting type 2 macrophages by EGFR-targeting antibody cetuximab. Clin Cancer Res.

[190] Shojaei F, Wu X, Qu X (2009). G-CSF-initiated myeloid cell mobilization and angiogenesis mediate tumor refractoriness to anti-VEGF therapy in mouse models. Proc Natl Acad Sci USA.

[191] Priceman SJ, Sung JL, Shaposhnik Z (2010). Targeting distinct tumor-infiltrating myeloid cells by inhibiting CSF-1 receptor: combating tumor evasion of antiangiogenic therapy. Blood.

[192] Sumida K, Wakita D, Narita Y (2012). Anti-IL-6 receptor mAb eliminates myeloid-derived suppressor cells and inhibits tumor growth by enhancing T-cell responses. Eur J Immunol.

[193] Melani C, Sangaletti S, Barazzetta FM, Werb Z, Colombo MP (2007). Amino-biphosphonate-mediated MMP-9 inhibition breaks the tumor-bone marrow axis responsible for myeloid-derived suppressor cell expansion and macrophage infiltration in tumor stroma. Cancer Res.

[194] Serafini P, Meckel K, Kelso M (2006). Phosphodiesterase-5 inhibition augments endogenous antitumor immunity by reducing myeloid-derived suppressor cell function. J Exp Med.

[195] Zhang J, Patel L, Pienta KJ (2010). CC chemokine ligand 2 (CCL2) promotes prostate cancer tumorigenesis and metastasis. Cytokine Growth Factor Rev.

[196] Yang L, Huang J, Ren X (2008). Abrogation of TGF beta signaling in mammary carcinomas recruits Gr-1+ CD11b+ myeloid cells that promote metastasis. Cancer Cell.

[197] Affara NI, Ruffell B, Medler TR (2014). B cells regulate macrophage phenotype and response to chemotherapy in squamous carcinomas. Cancer Cell.

[198] Watkins SK, Egilmez NK, Suttles J, Stout RD (2007). IL-12 rapidly alters the functional profile of tumor-associated and tumor-infiltrating macrophages in vitro and in vivo. J Immunol.

[199] Park S, Jiang Z, Mortenson ED (2010). The therapeutic effect of anti-HER2/neu antibody depends on both innate and adaptive immunity. Cancer Cell.

[200] Chao MP, Alizadeh AA, Tang C (2010). Anti-CD47 antibody synergizes with rituximab to promote phagocytosis and eradicate non-Hodgkin lymphoma. Cell.

[201] D’Alincourt Salazar M, Manuel ER, Tsai W (2016). Evaluation of innate and adaptive immunity contributing to the antitumor effects of PD1 blockade in an orthotopic murine model of pancreatic cancer. Oncoimmunology.

[202] Beatty GL, Chiorean EG, Fishman MP (2011). CD40 agonists alter tumor stroma and show efficacy against pancreatic carcinoma in mice and humans. Science.

[203] Laoui D, Van Overmeire E, Movahedi K (2011). Mononuclear phagocyte heterogeneity in cancer: different subsets and activation states reaching out at the tumor site. Immunobiology.

[204] De Palma M, Mazzieri R, Politi LS (2008). Tumor-targeted interferon-alpha delivery by Tie2-expressing monocytes inhibits tumor growth and metastasis. Cancer Cell.

[205] Muthana M, Rodrigues S, Chen YY (2013). Macrophage delivery of an oncolytic virus abolishes tumor regrowth and metastasis after chemotherapy or irradiation. Cancer Res.

[206] Olive KP, Jacobetz MA, Davidson CJ (2009). Inhibition of Hedgehog signaling enhances delivery of chemotherapy in a mouse model of pancreatic cancer. Science.

[207] Kindler HL, Niedzwiecki D, Hollis D (2010). Gemcitabine plus bevacizumab compared with gemcitabine plus placebo in patients with advanced pancreatic cancer: phase III trial of the Cancer and Leukemia Group B (CALGB 80303). J Clin Oncol.

[208] Kindler HL, Ioka T, Richel DJ (2011). Axitinib plus gemcitabine versus placebo plus gemcitabine in patients with advanced pancreatic adenocarcinoma: a double-blind randomised phase 3 study. Lancet Oncol.

[209] Ko AH, LoConte N, Tempero MA (2016). A phase I study of FOLFIRINOX plus IPI-926, a hedgehog pathway inhibitor, for advanced pancreatic adenocarcinoma. Pancreas.

[210] Jones LE, Humphreys MJ, Campbell F, Neoptolemos JP, Boyd MT (2004). Comprehensive analysis of matrix metalloproteinase and tissue inhibitor expression in pancreatic cancer: increased expression of matrix metalloproteinase-7 predicts poor survival. Clin Cancer Res.

[211] Moore MJ, Hamm J, Dancey J (2003). Comparison of gemcitabine versus the matrix metalloproteinase inhibitor BAY 12-9566 in patients with advanced or metastatic adenocarcinoma of the pancreas: a phase III trial of the National Cancer Institute of Canada Clinical Trials Group. J Clin Oncol.

[212] Ozdemir BC, Pentcheva-Hoang T, Carstens JL (2014). Depletion of carcinoma-associated fibroblasts and fibrosis induces immunosuppression and accelerates pancreas cancer with reduced survival. Cancer Cell.

[213] Rhim AD, Oberstein PE, Thomas DH (2014). Stromal elements act to restrain, rather than support pancreatic ductal adenocarcinoma. Cancer Cell.

[214] Gore J, Korc M (2014). Pancreatic cancer stroma: friend or foe?. Cancer Cell.

[215] Sherman MH, Yu RT, Engle DD (2014). Vitamin D receptor-mediated stromal reprogramming suppresses pancreatitis and enhances pancreatic cancer therapy. Cell.

[216] Froeling FE, Feig C, Chelala C (2011). Retinoic acid-induced pancreatic stellate cell quiescence reduces paracrine Wnt-beta-catenin signaling to slow tumor progression. Gastroenterology.

[217] Provenzano PP, Cuevas C, Chang AE (2012). Enzymatic targeting of the stroma ablates physical barriers to treatment of pancreatic ductal adenocarcinoma. Cancer Cell.

[218] Jacobetz MA, Chan DS, Neesse A (2013). Hyaluronan impairs vascular function and drug delivery in a mouse model of pancreatic cancer. Gut.

[219] Strimpakos AS, Saif MW (2013). Update on phase I studies in advanced pancreatic adenocarcinoma. Hunting in darkness?. JOP.

[220] Mediavilla-Varela M, Boateng K, Noyes D, Antonia SJ (2016). The anti-fibrotic agent pirfenidone synergizes with cisplatin in killing tumor cells and cancer-associated fibroblasts. BMC Cancer..

