# Supplementary material for: Chemotherapy and tumor microenvironment of pancreatic cancer
Source: Cancer Cell Int. 2017 Jul 5;17:68. doi: 10.1186/s12935-017-0437-3 (PMC5498917; doi:10.1186/s12935-017-0437-3)
Supplement: Supplementary file 1 — Additional file 1: Table S1. The chronological list of landmark events of chemotherapy in pancreatic cancer from 2000. Table S2. The pro-tumoral and anti-tumoral remodeling effects of chemotherapy on TME. Table S3. Tumor microenvironment targeting therapies of pancreatic cancer. [file 12935_2017_437_MOESM1_ESM.docx]

**Additional material**

**Chemotherapy** **And Tumor Microenvironment Of Pancreatic Cancer**

Qiaofei Liu, Quan Liao*, Yupei Zhao*

Department of General Surgery, Peking Union Medical College Hospital, Peking Union Medical College & Chinese Academy of Medical Sciences, Beijing 100730, China

Correspondence should be addressed to

Quan Liao, [lqpumc@126.com](mailto:lqpumc@126.com) and Yupei Zhao, [zhao8028@263.net](mailto:zhao8028@263.net)

**Table.1 The chronological list of landmark events of chemotherapy in pancreatic cancer from 2000**

| **Neoadjuvant Chemotherapy** | | | |
| --- | --- | --- | --- |
| **Date** | **Journal** | **Type of Trial** | **Main Results** |
| 2015[[1](#_ENREF_1)] | Ann Surg | Retrospective,  Single center | Compared with no neoadjuvant treatment, FOLFIRINOX resulted in lower operative morbidity (36% vs 63%), decrease in lymph node positivity (35% vs 79%), perineural invasion (72% vs 95%), and increase in overall survival for locally advanced and borderline resectable pancreatic cancer. |
| 2015[[2](#_ENREF_2)] | Strahlenther  Onkol | Prospective, Randomized,  Single center | This was the first randomized trial for neoadjuvant chemoradiotherapy in pancreatic cancer, however it was terminated due to slow recruiting. |
| **Adjuvant Chemotherapy** | | | |
| **Date** | **Journal** | **Type of Trial** | **Main Results** |
| 2001[[3](#_ENREF_3)] | Lancet | Prospective, Randomized,  Multicenter | Adjuvant chemoradiotherapy showed no survival benefit(median survival,15.5 months vs 16.1 months) but adjuvant chemotherapy(folinic acid and fluorouracil) revealed survival benefit (median survival, 19.7 months vs 14.0 months),compared with observation. |
| 2004[[4](#_ENREF_4)] | N Engl J Med | Prospective, Randomized,  Multicenter | Fluororacil based adjuvant chemotherapy had a significant survival benefit(5-year overall survival ,21% vs 8%), whereas adjuvant chemoradiotherapy had a worse effect on survival(5-year overall survival, 10% vs 20%), compared with observation. |
| 2007[[5](#_ENREF_5)] | JAMA | Prospective, Randomized, Multicenter | Postoperative gemcitabine improved the estimated disease free survival at 3 years and 5 years(23.5% and 16.5% vs 7.5% and 5.5%) compared with observation. |
| 2010[[6](#_ENREF_6)] | JAMA | Prospective, Randomized, Multicenter | Adjuvant use of fluorouracil plus folinic acid had comparable results with gemcitabine(median survival, 23.0 months vs 23.6 months). |
| 2013[[7](#_ENREF_7)] | JAMA | Prospective, Randomized, Multicenter | The use of adjuvant gemcitabine compared with observation alone increased 5-year overall survival (20.7 months vs 10.4 months), 10-year overall survival (12.2 months vs 7.7months )and median disease-free survival(13.4 months vs 6.7 months). |
| 2016[[8](#_ENREF_8)] | Lancet | Prospective, Randomized, Multicenter | Adjuvant chemotherapy with S-1 achieved better 5-year overall survial compared with gemcitabine(44.1% vs 24.4%). |
| 2017[[9](#_ENREF_9)] | Lancet | Prospective, Randomized, Multicenter | The median overall survival of the patients after resection in the gemcitabine plus capecitabine group was 28.0 months compared with 25.5 months in the gemcitabine group. |
| **Pilliative Chemotherapy** | | | |
| **Date** | **Journal** | **Type of Trial** | **Main Results** |
| 2011[[10](#_ENREF_10)] | N Engl J Med | Prospective, Randomized, Multicenter | Compared with gemcitabine, FOLFIRINOX achieved better objective response rate(31.6% vs 9.4%), median progression free survival(6.4 months vs 3.3 months) and overall survival(11.1months vs 6.8 months) for metastatic pancreatic cancer. |
| 2013[[11](#_ENREF_11)] | N Engl J Med | Prospective, Randomized, Multicenter | In patients with metastatic pancreatic cancer, nab-paclitaxel plus gemcitabine significantly improved median overall survival(8.5 months vs 6.7 months), median progression-free survival(5.5 months vs 3.7 months), and response rate (23% vs 7%), compared with gemcitabine alone. |
| 2014[[12](#_ENREF_12)] | J Clin Oncol | Prospective, Randomized, Multicenter | Second-line OFF(oxaliplatin, folinic acid and fluorouracil) improved the results when compared with FF(folinic acid and fluorouracil) alone in patients with gemcitabine-refractory pancreatic cancer, in respect with median progression free survival(2.9 months vs 2.0 months) and median overall survival(5.9 months vs 3.3 months). |
| 2016[[13](#_ENREF_13)] | Lancet | Prospective, Randomized, Multicenter | NFF(nanoliposomal, folinic acid and fluorouracil) improved the median overall survival(6.1 months vs 4.9 months) when compared with FF(folinic acid and fluorouracil) alone in patients in metastatic pancreatic cancer after previous gemcitabine therapy. |

**Table.2 The pro-tumoral and anti-tumoral remodling effects of chemotherapy on TME**

| Treatment | Drug Type | Cancer | Tumor-supporting effects | Tumor-suppression effects |
| --- | --- | --- | --- | --- |
| Gemcitabine | Cytotoxic drug | Lung, breast cancer(mice);  EL-4 lymphoma(mice);  Pancreatic cancer  (human and mice) | 1.Promotion of M2 polarization[[14](#_ENREF_14)];  2.Induction of MDSC[[15](#_ENREF_15)];  3.Induction of Th17 response by MDSC[[16](#_ENREF_16)];  4.Induction of drug metalolizing enzyme in TAM[[17](#_ENREF_17)]; | 1.Inbiton of proliferation of MDSC[[18](#_ENREF_18), [19](#_ENREF_19)];  2.Depletion of Treg cells[[20](#_ENREF_20)]. |
| Doxorubicin | Cytotoxic drug | Breast cancer  (mice and human);  Leukemia  (mouse or human) | 1.Incrase of protease activity of TAMs[[21](#_ENREF_21)] | 1.Inhibition of proliferation of MDSC[[22](#_ENREF_22)];  2.Promotion of recruitment and differentiation of MDSC into APCs[[23](#_ENREF_23)];  3.Induction of immunogenic death of cancer cells[[24](#_ENREF_24)]; |
| Cyclophosphamide | Cytotoxic drug | Leukemia(mice) |  | 1.Recruitment and activation of macrophages. |
| 5-fluorouracil | Cytotoxic drug | Thymoma(mice);  Pancreatic cancer(human) | 1.Induction of MDSC[[15](#_ENREF_15)];  2.Activation of Th17 cells by secreting inflammasome-IL1β in MDSC [[16](#_ENREF_16)];  3.Activation of CAFs to maintain colorectal cancer initiating cells by IL-17A[[25](#_ENREF_25)]. | 1.Inhibition of MDSC expansion[[26](#_ENREF_26)];  2. Depletion of Treg cells[[27](#_ENREF_27)]. |
| Docetaxel | Cytotoxic drug | Breast cancer(mice) |  | 1.Inhibition of MDSC expansion, and polarization to M1 phenotype[[28](#_ENREF_28)]; |
| Trabectedin | Cytotoxic drug | Lung cancer, ovarian cancer, soft tissue sarcoma (mouse and human) |  | 1.Depletion of MO-MDSCs and TAMs[[29](#_ENREF_29), [30](#_ENREF_30)]; |
| Paclitaxel | Cytotoxic drug | Breast cancer(mice ) | 1.Recruitment of TAM[[31](#_ENREF_31)];  2.Increase of protease activity of TAMs[[21](#_ENREF_21)]; |  |
| Etoposide | Cytotoxic drug | Breast cancer(human) | 1.Increase of protease activity of TAMs[[21](#_ENREF_21)] |  |
| Platinum | Cytotoxic drug | Cervical cancer(human) | 1.Promotion of M2[[32](#_ENREF_32)]; |  |
| Cisplatinum | Cytotoxic drug | Colon cancer, lung cancer(human) | 1.Induction of M2 to maintain cancer stem cells[[33](#_ENREF_33)]; |  |
| Imatinib | Signal inhibitor | GIST(human and mouse) | 1. Promotion of M2[[34](#_ENREF_34)] |  |
| TKI(sunitinib) | Signal inhibitor | Renal clear carcinoma(mice) |  | 1.Inhibition of MDSC expansion[[35](#_ENREF_35)] |
| Sorafenib | Tyrose kinase inhibitor | HCC(human and mice) | 1.Recruitemnt of TAMs to promote angiogensis[[36](#_ENREF_36)]; | 2.Conversion of M2 polarization and activation of NK cells[[37](#_ENREF_37)]. |
| Neutralizing antibody to Kit | Anti-Kit | Colon cancer(mice) |  | 1.Inhibition of proliferation of MDSCs[[38](#_ENREF_38)] |
| Anibodies agaist VEGF | Anti-VEGF | Metastatic RCC(human) | 1.Recruitment of CD11b+ cells or TAMs by induced-hypoxia reaction[[39](#_ENREF_39)]; | 1. Weak inhibition of proliferation of MDSCs[[40](#_ENREF_40)]; |
| Trastuzumab | Anti-HER-2 | Breast cancer(human) |  | 1.Induction of M1 polarized TAMs[[41](#_ENREF_41)] |
| Rituximab | Anti-CD20 | B cell lymphoma(human) |  | 1.Induction of M1 polarized TAMs[[42](#_ENREF_42)] |
| Cetuximab | Anti-EGFR | Colorectal cancer(human) | 1.Promotion of M2 polarized TAMs[[43](#_ENREF_43)] |  |
| Antibodies agaist CTLA4 | Anti-CTLA4 | Colorectal cancer(mice) |  | 1.Induction of M1 polarized TAMs, depletion of Treg cells[[44](#_ENREF_44), [45](#_ENREF_45)] |

Table.3 Tumor microenvironment targeting therapies of pancreatic cancer

| Study | Type | Target | Drug | Results |
| --- | --- | --- | --- | --- |
| Yao et al.,2017[[46](#_ENREF_46)] | Experimental | TAM | IL-27 | IL-27 inhibits M2 polarization to enhance the efficacy of gemcitabine. |
| Prakash, et al., 2016[[47](#_ENREF_47)] | Experimental | TAM | LPS, IFN-γ | LPS alone or combination with IFN-γ recaliberates cancer-induced M2 and reduce the tumor growth. |
| Liu et al., 2016[[14](#_ENREF_14)] | Experimental | TAM, MDSC | Aspirin, Atrovastatin | Aspirin retards the pancreatic cancerogenesis and enhances the effacicy of gemitabine by inhibition of M2 and MDSC. However, Atorvastatin promotes M2 polarization. |
| Incio, et al. 2015[[48](#_ENREF_48)] | Experimental | PSC, TAM | Metformin | Metformin alleviates the fibro-inflammatory microenvironment of pancreatic cancer by reprogramming PSCs and TAMs to reduce disease progression. |
| Zhu, et al., 2014[[49](#_ENREF_49)] | Experimental | MDSC, TAM | CSF1/CSFR1 blockade | PD1 and CTLA4 antagonists in combination with CSF1R blockade elicits tumor regression. |
| Griesmann, et al., 2016[[50](#_ENREF_50)] | Experimental | TAM | Liposomal clodronate | Depletion of macrophages by liposomal clodronate markedly reduces metastasis of pancreatic cancer and is associated with impaired angiogenesis and reduced Treg cells. |
| Hiroshima, et al., 2014[[51](#_ENREF_51)] | Experimental | TAM | Zoledronic acid (ZA) | Depletion of TAM by ZA inhibits tumor growth and metastasis of human pancreatic cancer. |
| Bayne, et al., 2012[[52](#_ENREF_52)] | Experimental | TAM | CSF antibody | CSF antibody inhibits expansion and recruitment of TAMs to reduce tumor progression. |
| Beatty, et al, 2011[[53](#_ENREF_53), [54](#_ENREF_54)] | Experimental, phase I clinical trial | TAM | CD 40 agonist | Activation of TAMs by CD40 exerts anti-tumor activities and improves the efficacy of gemcitabine. |
| Nywening, et al., 2016[[55](#_ENREF_55)] | Phase 1b clinical trail | TAM | CCR2 inhibitor | CCR2 inhibition with FOLFIRINOX in borderline resectable and locally advanced pancreatic cancer patients is safe and tolerable. |
| Karakhanov, et al., 2015[[56](#_ENREF_56)] | Experimental | MDSC | phosphodiesterase-5 inhibitor(sildenafil) | Sildenafil prolongs the survival of PDAC-bearing female mice, due to the decrease of MDSC and in the systemic VEGF level. |
| Chronopoulos, et al.,2016[[57](#_ENREF_57)] | Experimental | PSC | an active metabolite of vitamin A(ATRA) | ATRA restores quiescence of PSC via a retinoic acid receptor beta (RAR-beta)-dependent downregulation of actomyosin (MLC-2) to inhibit local cancer cell invasion. |
| Horioka, et al., 2016[[58](#_ENREF_58)] | Experimental | PCS | Inhibition of CD51 | Inhibition of CD51 of PCSs retards the growth of cancer cells. |
| Masamune, et al., 2013[[59](#_ENREF_59)] | Experimental | PSC | Angiotensin II type I receptor blocker (olmesartan) | Olmesartan decreases cell growth and type I collagen production in PSCs to inhibit tumor growth. |
| Kozono, et al., 2013[[60](#_ENREF_60)] | Experimental | PSC | Antifibrotic agent (prifenidone) | Prifenidone improves the efficacy of gemcitabine by inhibition of PSCs. |
| Guan, et al.,2013[[61](#_ENREF_61)] | Experimental | CAF | Retinoic acid(RA) | RA restores quiescence of PSC to inhibit EMT of cancer cells. |
| Moatassim-Billah, et al., 2016[[62](#_ENREF_62)] | experimental | CAF | Somatostatin analog (SOM230) | SOM230 induces quiescence of CAFs to inhibit tumor growth and abrogate metastasis. |
| Oliver, et al., 2009[[63](#_ENREF_63)] | experimental | CAF | sonic hedgehog (shh) inhibitor | Depletion of CAF by SHH inhibitor results in increased vascularization and more effective drug delivery with improve overall survival. |
| Ko, et al., 2016[[64](#_ENREF_64)] | Phase I clinial trial | CAF | Shh inhibitor(IPI-926) | The study was closed early because IPI-926 in combination with folfirinox indicated a shorter survival. |
| Ozdemir, et al., 2014[[65](#_ENREF_65)] | Experimental | CAF | Genetic depletion | Genetic depletion of CAFs leads to more aggressive pancreatic cancer and the pancreatic cancer does not respond to gemcitabine. |
| Sherman, et al., 2014[[66](#_ENREF_66)] | Experimental | CAF | Vitamin D analogue(calcipotriol) | Calcipotriol induces quiescence of CAF to improve the efficacy of gemcitabine by elevation the intratumoral drug concentration. |
| Bramahall, et al., 2002[[67](#_ENREF_67)] | Phase II clinical trial | ECM | MMP inhibitor (Marimastat) | The combination of marimastat with gemcitabine does not show any benefit. |
| Moore, et al., 2003[[68](#_ENREF_68)] | Phase III clinical trial | ECM | MMP inhibitor(Bay 12-9566) | The clinical trial was terminated because Bay 12-9566 was inferior to gemcitabine alone. |
| Strimpakoe, et al., 2013[[69](#_ENREF_69)] | Phase I clinical trial | Hyaluronan | PEGPH 20 | Phase I showed no significant toxicity when it was used with gemcitabine. |
| Kindler, et al., 2010[[70](#_ENREF_70)] | Phase III clinical trial | VEGF-A | VEGF-A antibody (bevacizumab) | Bevacizumab in combination with gemcitabine does not improve survival. |
| Kindler, et al., 2012[[71](#_ENREF_71)] | Phase III clinical trial | VEGF receptors | VEGF receptor inhibitor (oxitinib) | Oxitinib in combination with gemcitabine did not improve overall survival. |
| Brahmer, et al., 2012[[72](#_ENREF_72)] | Phase I clinical trial | PDL1 | PDL1 inhibitor(BMS-936559) | BMS-936559 does not show any objective response, however it wa safe. |
| Royal, et al., 2010[[73](#_ENREF_73)] | Phase II clinical trial | CTLA-4 | CTLA-4 inhibitor(lpilimumab) | Lpilimumab alone does not show an acceptable response, however it was safe. |

**References**

1. Ferrone CR, Marchegiani G, Hong TS, et al: Radiological and surgical implications of neoadjuvant treatment with FOLFIRINOX for locally advanced and borderline resectable pancreatic cancer. Ann Surg 2015, 261(1):12-17.

2. Golcher H, Brunner TB, Witzigmann H, et al: Neoadjuvant chemoradiation therapy with gemcitabine/cisplatin and surgery versus immediate surgery in resectable pancreatic cancer. Strahlenther Onkol 2015, 191(1):7-16.

3. Neoptolemos JP, Dunn JA, Stocken DD, et al: Adjuvant chemoradiotherapy and chemotherapy in resectable pancreatic cancer: a randomised controlled trial. Lancet 2001, 358(9293):1576-1585.

4. Neoptolemos JP, Stocken DD, Friess H, et al: A randomized trial of chemoradiotherapy and chemotherapy after resection of pancreatic cancer. N Engl J Med 2004, 350(12):1200-1210.

5. Oettle H, Post S, Neuhaus P, et al: Adjuvant chemotherapy with gemcitabine vs observation in patients undergoing curative-intent resection of pancreatic cancer - A Randomized controlled trial. Jama 2007, 297(3):267-277.

6. Neoptolemos JP, Stocken DD, Bassi C, et al: Adjuvant chemotherapy with fluorouracil plus folinic acid vs gemcitabine following pancreatic cancer resection: a randomized controlled trial. Jama 2010, 304(10):1073-1081.

7. Oettle H, Neuhaus P, Hochhaus A, et al: Adjuvant Chemotherapy With Gemcitabine and Long-term Outcomes Among Patients With Resected Pancreatic Cancer The CONKO-001 Randomized Trial. Jama 2013, 310(14):1473-1481.

8. Uesaka K, Boku N, Fukutomi A, et al: Adjuvant chemotherapy of S-1 versus gemcitabine for resected pancreatic cancer: a phase 3, open-label, randomised, non-inferiority trial (JASPAC 01). Lancet 2016, 388(10041):248-257.

9. Neoptolemos JP, Palmer DH, Ghaneh P, et al: Comparison of adjuvant gemcitabine and capecitabine with gemcitabine monotherapy in patients with resected pancreatic cancer (ESPAC-4): a multicentre, open-label, randomised, phase 3 trial. Lancet 2017, 389(10073):1011-1024.

10. Conroy T, Desseigne F, Ychou M, et al: FOLFIRINOX versus gemcitabine for metastatic pancreatic cancer. N Engl J Med 2011, 364(19):1817-1825.

11. Von Hoff DD, Ervin T, Arena FP, et al: Increased survival in pancreatic cancer with nab-paclitaxel plus gemcitabine. N Engl J Med 2013, 369(18):1691-1703.

12. Oettle H, Riess H, Stieler JM, et al: Second-line oxaliplatin, folinic acid, and fluorouracil versus folinic acid and fluorouracil alone for gemcitabine-refractory pancreatic cancer: outcomes from the CONKO-003 trial. J Clin Oncol 2014, 32(23):2423-2429.

13. Wang-Gillam A, Li CP, Bodoky G, et al: Nanoliposomal irinotecan with fluorouracil and folinic acid in metastatic pancreatic cancer after previous gemcitabine-based therapy (NAPOLI-1): a global, randomised, open-label, phase 3 trial. Lancet 2016, 387(10018):545-557.

14. Liu Q, Li Y, Niu Z, Zong Y, Wang M, Yao L, et al: Atorvastatin (Lipitor) attenuates the effects of aspirin on pancreatic cancerogenesis and the chemotherapeutic efficacy of gemcitabine on pancreatic cancer by promoting M2 polarized tumor associated macrophages. J Exp Clin Cancer Res: CR 2016, 35:33.

15. Takeuchi S, Baghdadi M, Tsuchikawa T, et al: Chemotherapy-Derived Inflammatory Responses Accelerate the Formation of Immunosuppressive Myeloid Cells in the Tissue Microenvironment of Human Pancreatic Cancer. Cancer Res 2015, 75(13):2629-2640.

16. Bruchard M, Mignot G, Derangere V, et al: Chemotherapy-triggered cathepsin B release in myeloid-derived suppressor cells activates the Nlrp3 inflammasome and promotes tumor growth. Nat Med 2013, 19(1):57-64.

17. Weizman N, Krelin Y, Shabtay-Orbach A,et al: Macrophages mediate gemcitabine resistance of pancreatic adenocarcinoma by upregulating cytidine deaminase. Oncogene 2014, 33(29):3812-3819.

18. Suzuki E, Kapoor V, Jassar AS, Kaiser LR, Albelda SM: Gemcitabine selectively eliminates splenic Gr-1+/CD11b+ myeloid suppressor cells in tumor-bearing animals and enhances antitumor immune activity. Clin Cancer Res 2005, 11(18):6713-6721.

19. Liu QF, Li Y, Niu ZY, et al, Zhao YP: Atorvastatin (Lipitor) attenuates the effects of aspirin on pancreatic cancerogenesis and the chemotherapeutic efficacy of gemcitabine on pancreatic cancer by promoting M2 polarized tumor associated macrophages. J Exp Clin Canc Res 2016, 35.

20. Shevchenko I, Karakhanova S, Soltek S,et al: Low-dose gemcitabine depletes regulatory T cells and improves survival in the orthotopic Panc02 model of pancreatic cancer. Int J Cancer 2013, 133(1):98-107.

21. Shree T, Olson OC, Elie BT,et al: Macrophages and cathepsin proteases blunt chemotherapeutic response in breast cancer. Genes Dev 2011, 25(23):2465-2479.

22. Diaz-Montero CM, Salem ML, Nishimura MI, Garrett-Mayer E, Cole DJ, Montero AJ: Increased circulating myeloid-derived suppressor cells correlate with clinical cancer stage, metastatic tumor burden, and doxorubicin-cyclophosphamide chemotherapy. Cancer Immunol Immunother 2009, 58(1):49-59.

23. Mantovani A, Biswas SK, Galdiero MR, Sica A, Locati M: Macrophage plasticity and polarization in tissue repair and remodelling. J Pathol 2013, 229(2):176-185.

24. De Palma M, Lewis CE: Macrophage regulation of tumor responses to anticancer therapies. Cancer cell 2013, 23(3):277-286.

25. Lotti F, Jarrar AM, Pai RK, et al: Chemotherapy activates cancer-associated fibroblasts to maintain colorectal cancer-initiating cells by IL-17A. J Exp Med 2013, 210(13):2851-2872.

26. Vincent J, Mignot G, Chalmin F,et al: 5-Fluorouracil selectively kills tumor-associated myeloid-derived suppressor cells resulting in enhanced T cell-dependent antitumor immunity. Cancer Res 2010, 70(8):3052-3061.

27. Hao YB, Yi SY, Ruan J, Zhao L, Nan KJ: New insights into metronomic chemotherapy-induced immunoregulation. Cancer Lett 2014, 354(2):220-226.

28. Kodumudi KN, Woan K, Gilvary DL, Sahakian E, Wei S, Djeu JY: A Novel Chemoimmunomodulating Property of Docetaxel: Suppression of Myeloid-Derived Suppressor Cells in Tumor Bearers. Clin Cancer Res 2010, 16(18):4583-4594.

29. Germano G, Frapolli R, Belgiovine C, et al: Role of Macrophage Targeting in the Antitumor Activity of Trabectedin. Cancer cell 2013, 23(2):249-262.

30. Germano G, Frapolli R, Belgiovine C, et al: Role of macrophage targeting in the antitumor activity of trabectedin. Cancer cell 2013, 23(2):249-262.

31. DeNardo DG, Brennan DJ, Rexhepaj E, et al: Leukocyte complexity predicts breast cancer survival and functionally regulates response to chemotherapy. Cancer Discov 2011, 1(1):54-67.

32. Dijkgraaf EM, Heusinkveld M, Tummers B,et al: Chemotherapy Alters Monocyte Differentiation to Favor Generation of Cancer-Supporting M2 Macrophages in the Tumor Microenvironment. Cancer Res 2013, 73(8):2480-2492.

33. Jinushi M, Chiba S, Yoshiyama H,et al: Tumor-associated macrophages regulate tumorigenicity and anticancer drug responses of cancer stem/initiating cells. Proc Natl Acad Sci U S A 2011, 108(30):12425-12430.

34. Cavnar MJ, Zeng S, Kim TS et al: KIT oncogene inhibition drives intratumoral macrophage M2 polarization. J Exp Med 2013, 210(13):2873-2886.

35. van Cruijsen H, van der Veldt AAM, Vroling L, et al: Sunitinib-induced myeloid lineage redistribution in renal cell cancer patients: CD1c(+) dendritic cell frequency predicts progression-free survival. Clin Cancer Res 2008, 14(18):5884-5892.

36. Zhang CC, Yan ZM, Zhang Q, et al: PF-03732010: A Fully Human Monoclonal Antibody against P-Cadherin with Antitumor and Antimetastatic Activity. Clin Cancer Res 2010, 16(21):5177-5188.

37. Sprinzl MF, Reisinger F, Puschnik A, et al: Sorafenib perpetuates cellular anticancer effector functions by modulating the crosstalk between macrophages and natural killer cells. Hepatology 2013, 57(6):2358-2368.

38. Pan PY, Wang GX, Yin B, et al: Reversion of immune tolerance in advanced malignancy: modulation of myeloid-derived suppressor cell development by blockade of stem-cell factor function. Blood 2008, 111(1):219-228.

39. Lu-Emerson C, Snuderl M, Kirkpatrick ND, et al: Increase in tumor-associated macrophages after antiangiogenic therapy is associated with poor survival among patients with recurrent glioblastoma. Neuro Oncol 2013, 15(8):1079-1087.

40. Shojaei F, Wu XM, Malik AK, et al: Tumor refractoriness to anti-VEGF treatment is mediated by CD11b(+)Gr1(+) myeloid cells. Nat Biotechnol 2007, 25(8):911-920.

41. Sliwkowski MX, Mellman I: Antibody therapeutics in cancer. Science 2013, 341(6151):1192-1198.

42. Furness AJ, Vargas FA, Peggs KS, Quezada SA: Impact of tumour microenvironment and Fc receptors on the activity of immunomodulatory antibodies. Trends Immunol 2014, 35(7):290-298.

43. Pander J, Heusinkveld M, van der Straaten T, et al: Activation of tumor-promoting type 2 macrophages by EGFR-targeting antibody cetuximab. Clin Cancer Res 2011, 17(17):5668-5673.

44. Simpson TR, Li F, Montalvo-Ortiz W, Sepulveda MA, et al: Fc-dependent depletion of tumor-infiltrating regulatory T cells co-defines the efficacy of anti-CTLA-4 therapy against melanoma. J Exp Med 2013, 210(9):1695-1710.

45. Selby MJ, Engelhardt JJ, Quigley M, et al: Anti-CTLA-4 antibodies of IgG2a isotype enhance antitumor activity through reduction of intratumoral regulatory T cells.Cancer Immunol Res 2013, 1(1):32-42.

46. Yao L, Wang M, Niu Z, et al: Interleukin-27 inhibits malignant behaviors of pancreatic cancer cells by targeting M2 polarized tumor associated macrophages. Cytokine 2017, 89:194-200.

47. Prakash H, Nadella V, Singh S, Schmitz-Winnenthal H: CD14/TLR4 priming potentially recalibrates and exerts anti-tumor efficacy in tumor associated macrophages in a mouse model of pancreatic carcinoma. Sci Rep 2016, 6:31490.

48. Incio J, Suboj P, Chin SM, et al: Metformin Reduces Desmoplasia in Pancreatic Cancer by Reprogramming Stellate Cells and Tumor-Associated Macrophages. PloS one 2015, 10(12):e0141392.

49. Zhu Y, Knolhoff BL, Meyer MA,et al: CSF1/CSF1R blockade reprograms tumor-infiltrating macrophages and improves response to T-cell checkpoint immunotherapy in pancreatic cancer models. Cancer Res 2014, 74(18):5057-5069.

50. Griesmann H, Drexel C, Milosevic N, et al: Pharmacological macrophage inhibition decreases metastasis formation in a genetic model of pancreatic cancer. Gut 2016.

51. Hiroshima Y, Maawy A, Hassanein MK, et al: The tumor-educated-macrophage increase of malignancy of human pancreatic cancer is prevented by zoledronic acid. PloS one 2014, 9(8):e103382.

52. Bayne LJ, Beatty GL, Jhala N, et al: Tumor-derived granulocyte-macrophage colony-stimulating factor regulates myeloid inflammation and T cell immunity in pancreatic cancer. Cancer cell 2012, 21(6):822-835.

53. Beatty GL, Chiorean EG, Fishman MP, et al: CD40 Agonists Alter Tumor Stroma and Show Efficacy Against Pancreatic Carcinoma in Mice and Humans. Science 2011, 331(6024):1612-1616.

54. Beatty GL, Torigian DA, Chiorean EG, et al: A phase I study of an agonist CD40 monoclonal antibody (CP-870,893) in combination with gemcitabine in patients with advanced pancreatic ductal adenocarcinoma. Clin Cancer Res 2013, 19(22):6286-6295.

55. Nywening TM, Wang-Gillam A, Sanford DE, et al: Targeting tumour-associated macrophages with CCR2 inhibition in combination with FOLFIRINOX in patients with borderline resectable and locally advanced pancreatic cancer: a single-centre, open-label, dose-finding, non-randomised, phase 1b trial. Lancet Oncol 2016, 17(5):651-662.

56. Karakhanova S, Link J, Heinrich M, et al: Characterization of myeloid leukocytes and soluble mediators in pancreatic cancer: importance of myeloid-derived suppressor cells. Oncoimmunology 2015, 4(4):e998519.

57. Chronopoulos A, Robinson B, Sarper M, et al: ATRA mechanically reprograms pancreatic stellate cells to suppress matrix remodelling and inhibit cancer cell invasion. Nat Commun 2016, 7:12630.

58. Horioka K, Ohuchida K, Sada M, et al: Suppression of CD51 in pancreatic stellate cells inhibits tumor growth by reducing stroma and altering tumor-stromal interaction in pancreatic cancer. Int J Oncol 2016, 48(4):1499-1508.

59. Masamune A, Hamada S, Kikuta K, et al: The angiotensin II type I receptor blocker olmesartan inhibits the growth of pancreatic cancer by targeting stellate cell activities in mice. Scand J Gastroenterol. 2013, 48(5):602-609.

60. Kozono S, Ohuchida K, Eguchi D, et al: Pirfenidone inhibits pancreatic cancer desmoplasia by regulating stellate cells. Cancer Res 2013, 73(7):2345-2356.

61. Guan J, Zhang H, Wen Z,et al: Retinoic acid inhibits pancreatic cancer cell migration and EMT through the downregulation of IL-6 in cancer associated fibroblast cells. Cancer Lett 2014, 345(1):132-139.

62. Moatassim-Billah S, Duluc C, Samain R, et al: Anti-metastatic potential of somatostatin analog SOM230: Indirect pharmacological targeting of pancreatic cancer-associated fibroblasts. Oncotarget 2016, 7(27):41584-41598.

63. Olive KP, Jacobetz MA, Davidson CJ, et al: Inhibition of Hedgehog signaling enhances delivery of chemotherapy in a mouse model of pancreatic cancer. Science 2009, 324(5933):1457-1461.

64. Ko AH, LoConte N, Tempero MA, et al: A Phase I Study of FOLFIRINOX Plus IPI-926, a Hedgehog Pathway Inhibitor, for Advanced Pancreatic Adenocarcinoma. Pancreas 2016, 45(3):370-375.

65. Ozdemir BC, Pentcheva-Hoang T, Carstens JL, et al: Depletion of carcinoma-associated fibroblasts and fibrosis induces immunosuppression and accelerates pancreas cancer with reduced survival. Cancer cell 2014, 25(6):719-734.

66. Sherman MH, Yu RT, et al: Vitamin D receptor-mediated stromal reprogramming suppresses pancreatitis and enhances pancreatic cancer therapy. Cell 2014, 159(1):80-93.

67. Bramhall SR, Schulz J, Nemunaitis J, Brown PD, Baillet M, Buckels JAC: A double-blind placebo-controlled, randomised study comparing gemcitabine and marimastat with gemcitabine and placebo as first line therapy in patients with advanced pancreatic cancer. Br J Cancer 2002, 87(2):161-167.

68. Moore MJ, Hamm J, Dancey J, et al: Comparison of gemcitabine versus the matrix metalloproteinase inhibitor BAY 12-9566 in patients with advanced or metastatic adenocarcinoma of the pancreas: A phase III trial of the National Cancer Institute of Canada Clinical Trials Group. J Clin Oncol 2003, 21(17):3296-3302.

69. Strimpakos AS, Saif MW: Update on phase I studies in advanced pancreatic adenocarcinoma. Hunting in darkness? JOP : JOP 2013, 14(4):354-358.

70. Kindler HL, Niedzwiecki D, Hollis D, et al: Gemcitabine plus bevacizumab compared with gemcitabine plus placebo in patients with advanced pancreatic cancer: phase III trial of the Cancer and Leukemia Group B (CALGB 80303). J Clin Oncol. 2010, 28(22):3617-3622.

71. Kindler HL, Ioka T, Richel DJ, et al: Axitinib plus gemcitabine versus placebo plus gemcitabine in patients with advanced pancreatic adenocarcinoma: a double-blind randomised phase 3 study. Lancet Oncol 2011, 12(3):256-262.

72. Brahmer JR, Tykodi SS, Chow LQ, et al: Safety and activity of anti-PD-L1 antibody in patients with advanced cancer. N Engl J Med 2012, 366(26):2455-2465.

73. Royal RE, Levy C, Turner K, et al: Phase 2 trial of single agent Ipilimumab (anti-CTLA-4) for locally advanced or metastatic pancreatic adenocarcinoma. J Immunother 2010, 33(8):828-833.
